# Discovery of potent histone deacetylase inhibitors with modified phenanthridine caps

**DOI:** 10.1080/14756366.2021.1892089

**Published:** 2021-03-05

**Authors:** Wenli Fan, Lin Zhang, Xuejiang Wang, Haiyong Jia, Lei Zhang

**Affiliations:** a Department of Medicinal Chemistry, School of Pharmacy, Weifang Medical University, Weifang, China; b Department of Pharmacology, School of Pharmacy, Weifang Medical University, Weifang, China

**Keywords:** Histone deacetylase, inhibitor, anticancer, phenanthridine, linker

## Abstract

In discovery of novel HDAC inhibitory with anticancer potency, pharmacophores of phenanthridine were introduced to the structure of HDAC inhibitors. Fatty and aromatic linkers were evaluated for their solubility and activity. Both enzyme inhibitory and *in vitro* antiproliferative (against U937 cells) screening results revealed better activities of compounds with aromatic linker than molecules with fatty linker. Compared with SAHA (IC_50_ values of 1.34, 0.14, 2.58, 0.67 and 18.17 µM), molecule **Fb-4** exhibited 0.87, 0.09, 0.32, 0.34 and 17.37 µM of IC_50_ values against K562, U266, MCF-7, U937 and HEPG2 cells, respectively. As revealed by cell cycle and apoptotic analysis, induction of G2/M phase arrest and apoptosis plays an important role in the inhibition of MCF-7 cells by **Fb-4**. Generally, a potent HDAC inhibitor was developed in the present study which could be utilised as a lead compound for further anticancer drug design.

## Introduction

Histone deacetylases (HDACs) are a group of enzymes that are responsible for the deacetylation ɛ-N-acetyl-lysine amino groups on histones and various non-histone proteins^1^^,^^2^. Until now, 18 isoforms of HDACs which are divided into 4 classes have been identified in human^3^^,^^4^. Class I HDACs (HDAC1, 2, 3 and 8) have been extensively studied in the field of anticancer drug development. Class II HDACs are subdivided into IIa (HDAC4, 5, 7 and 9) an IIb (HDAC6 and 10). Class III HDACs are a group of NAD^+^ dependent enzymes which are also termed sirtuins (Sirt1-7). There is only one member in class IV, HDAC11, which is the most recently identified and smallest known HDAC enzyme.

The modification of histone and non-histone proteins by HDACs and histone acetyl transferases (HATs) plays an important role in the regulation of cellular functions^5^. Overexpression and aberrant recruitment of HDACs (especially class I and II HDACs) are closely related to tumorigenesis and tumour development^6^^,^^7^. Inhibition of HDACs has been extensively investigated for the treatment of cancer. Various types of HDAC inhibitors have been designed and synthesised for the discovery of anticancer drugs^8^. The first US FDA approved HDAC inhibitor, Vorinostat (SAHA)^9^, has been utilised for the treatment of cutaneous T-cell lymphoma (CTCL). Following the success of SAHA, romidepsin (FK-228), belinostat (PXD101), and panobinostat (LBH589) were approved for the treatment of CTCL, peripheral T-cell lymphoma (PTCL), and multiple myeloma^10–12^, respectively.

There is a narrow tunnel in the active site of HDACs, and a zinc ion locates at the end of the tunnel. At the opening of the active site, according to different isoforms of HDACs, there are binding pockets with various shape and size. Generally, pharmacophores of HDAC inhibitors are consist of a zinc binding group (ZBG), a linker and a cap^8^. Class I, II and IV HDACs are zinc dependent enzymes, and there is a zinc ion in the active site of these HDACs. Therefore, ZBGs are needed in the structure of HDAC inhibitors for the binding of inhibitors to the active site. Cap region binds to the opening of the active site, aromatic groups in this region could form strong hydrophobic interactions with surrounding residues. Linker which is used to connect cap and ZBG can also interact with residues in the active site of HDACs.

**Figure 1.**
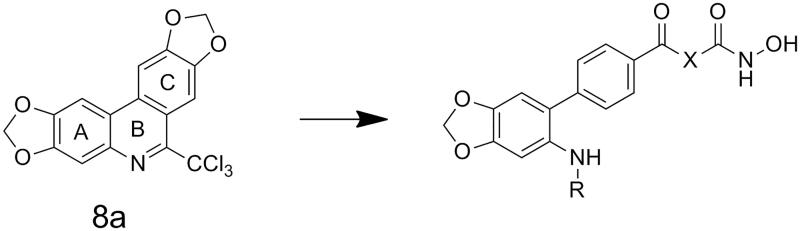
Design of novel HDAC inhibitors by introduction of phenanthridine pharmacophore to the cap region. X = –NH(CH_2_)_3_–, –NHC_6_H_4_–.

In our previous studies, a series of phenanthridine derivatives were developed with high anticancer activities by targeting DNA topoisomerase^13^. However, the synthesised molecules are extremely low at both aqueous solubility and liposolubility. Moreover, in the *in vivo* studies using xenograft nude mice model, molecule **8a** exhibited obvious hepatotoxicity. Considering the aromatic properties of phenanthridine structure, strong hydrophobic interactions could be formed between phenanthridine fragment and residues in the opening of HDAC active site. Therefore, in discovery of anticancer agents with improved solubility, activity and safety profiles, pharmacophores of phenanthridine was introduced to the cap region in the structure of HDACIs (Figure 1). By targeting HDACs, the toxicity of the designed molecules was considered to be reduced. To decrease the aromaticity of the designed compounds, the B ring in the phenanthridine structure was opened for the introduction of substituents. Hydroxamic acid group was utilised as zinc binding group, aromatic and fatty linkers were introduced, respectively. The synthesised target compounds were investigated in the enzyme inhibitory assay, *in vitro* antiproliferative screening, cell cycle and apoptosis test.

## Chemistry

**Scheme 1.**
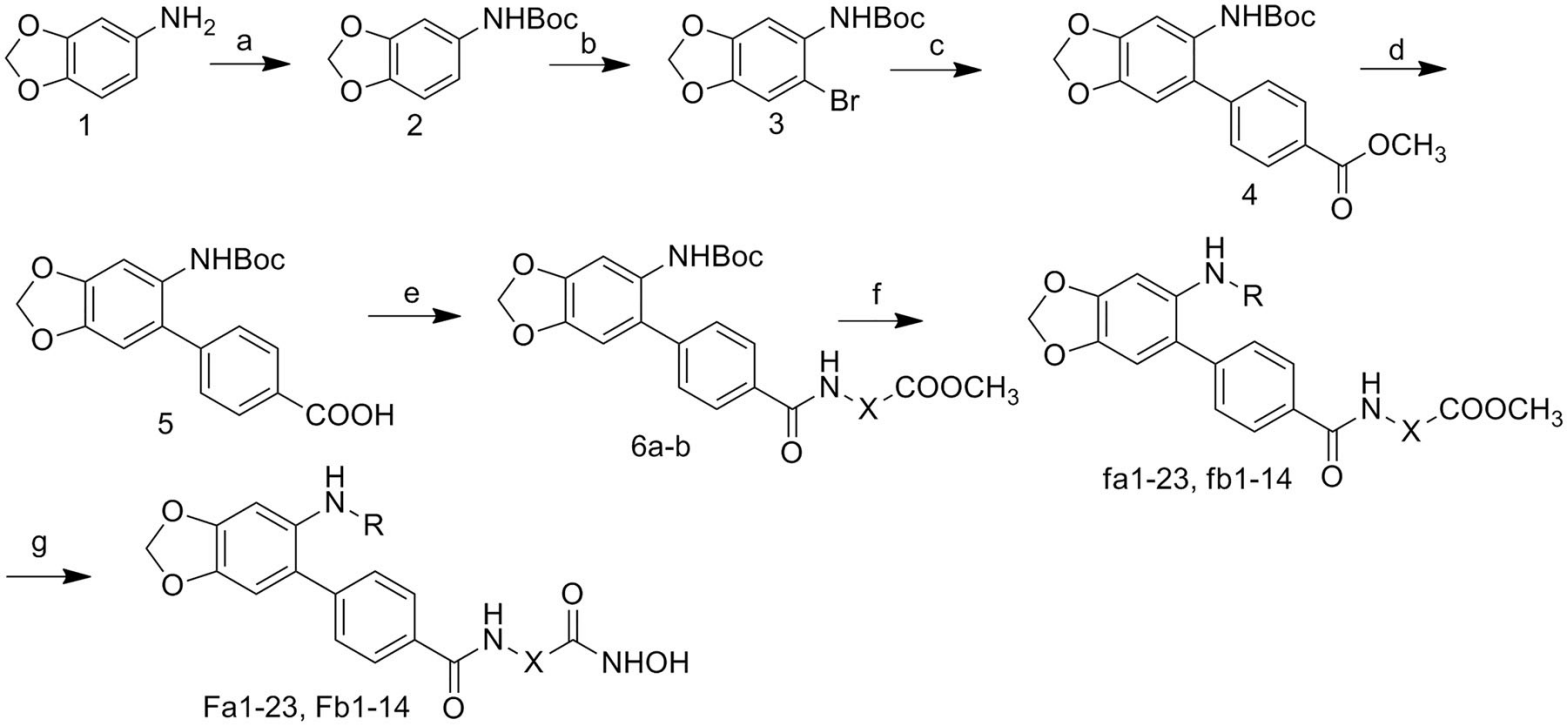
Reagents and conditions: (a) Boc_2_O, ACN, ice-bath; (b) NBS, ACN, rt; (c) K_2_CO_3,_ Trans-Dichlorobis(triphenyl-phosphine)Palladium(II), 1,4-dioxane, H_2_O, reflux; (d) 3 mol/l NaOH, MeOH, 40 °C; (e) TBTU, Et_3_N, DCM, ice-bath; (f) DCM, TFA; NaHCO_3_, THF, H_2_O, rt; (g) NH_2_OK, MeOH, rt.

The target molecules were synthesised as illustrated in scheme 1. At first, the amino group of the starting material benzo[*d*][1,3]dioxol-5-amine was protected by Boc group. The following bromine substitution was carried out to generate intermediate **3**. Preparation of intermediate 4 was performed by Suzuki coupling of intermediate **3** with 4-(Methoxycarbonyl)benzeneboronic acid. Deprotection of the carboxy group was accomplished by treatment of intermediate **4** under alkaline condition. Introduction of linkers was performed by condensation of intermediate **5** with corresponding amino carboxylate. Deprotection of amino group and following condensation were carried out to afford intermediate **fa1-23** and **fb1-14**. Target molecules (**Fa1-23**, **Fb1-14**) were synthesised by treatmentof corresponding intermediates with NH_2_OK in methanol.

## Results and discussions

### Enzymatic inhibition assay

The HDAC enzyme inhibitory activities of synthesised compounds were investigated by utilising Hela nucleus extract containing a mixture of HDAC isoforms. Percentage inhibitory rate was used to determine the activity of tested compounds (Tables 1 and 2). The **Fa** series compounds were firstly synthesised for the activity screening. The results revealed that all the compounds with fatty linker are less active compared with the positive control SAHA at concentration of 1 µM. To investigate whether aromatic linker could improve the inhibitory activities, structural modifications were performed on current molecules. The derived **Fb** series compounds with aromatic linker showed improved HDAC inhibitory activities compared with the **Fa** series molecules. Molecule **Fb-2**, **Fb-3** and **Fb-4** exhibited inhibitory rate of 52.30, 53.90 and 66.54, respectively, compared with SAHA (inhibitory rate of 56.23).

**Table 1.** Enzyme inhibitory and antiproliferative activities of Fa series compounds (percentage of inhibition at concentration of 1 µM). 
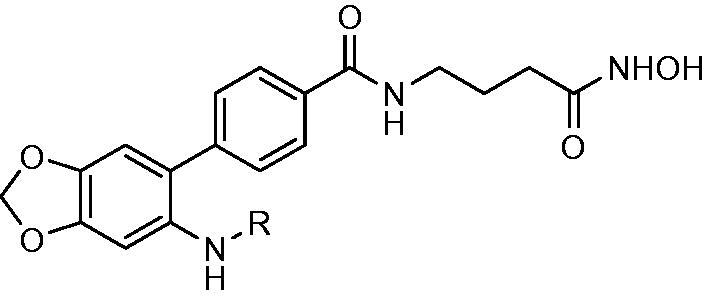

Compounds	R	HDACs^a^	U937^a^
Fa-1	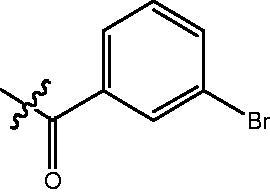	6.63 ± 1.28	21.04 ± 0.55
Fa-2	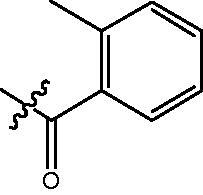	15.23 ± 3.23	19.16 ± 1.09
Fa-3	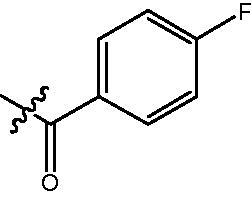	9.22 ± 2.28	19.36 ± 1.44
Fa-4	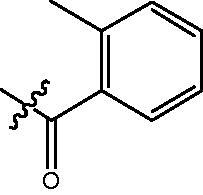	13.65 ± 4.04	18.21 ± 2.91
Fa-5	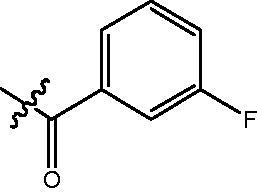	8.43 ± 1.74	19.91 ± 1.33
Fa-6	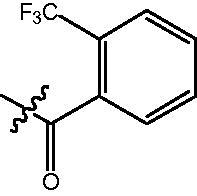	24.88 ± 4.88	15.01 ± 2.47
Fa-7	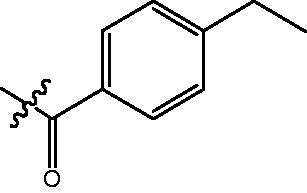	25.79 ± 5.79	22.17 ± 2.28
Fa-8	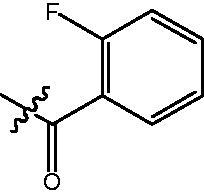	5.32 ± 1.72	19.89 ± 1.96
Fa-9	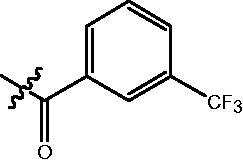	18.92 ± 1.30	20.45 ± 0.05
Fa-10	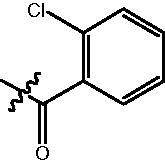	23.41 ± 1.95	23.02 ± 1.23
Fa-11	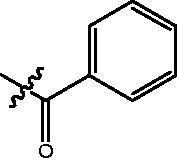	14.32 ± 2.19	25.60 ± 1.76
Fa-12	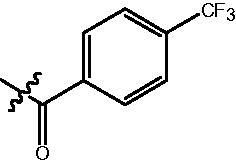	17.69 ± 3.52	48.49 ± 3.98
Fa-13	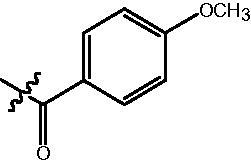	44.51 ± 0.22	88.89 ± 5.00
Fa-14	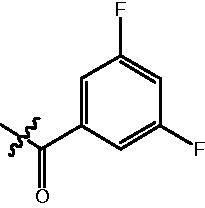	28.72 ± 4.74	9.84 ± 0.71
Fa-15	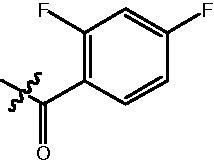	19.51 ± 4.10	6.76 ± 0.26
Fa-16	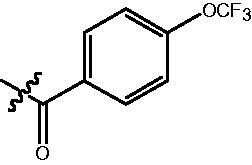	34.10 ± 1.02	16.13 ± 2.82
Fa-17	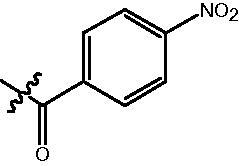	52.44 ± 6.17	13.42 ± 0.81
Fa-18	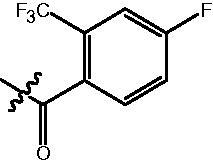	31.47 ± 3.47	10.29 ± 1.31
Fa-19	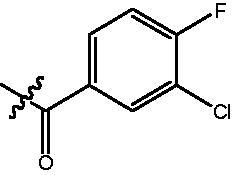	24.43 ± 2.90	15.79 ± 2.29
Fa-20	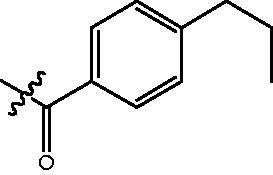	21.14 ± 2.55	12.15 ± 0.24
Fa-21	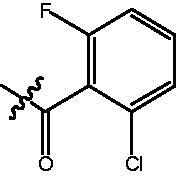	15.58 ± 3.25	11.24 ± 1.01
Fa-22	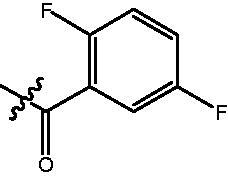	24.89 ± 4.89	4.73 ± 0.64
Fa-23	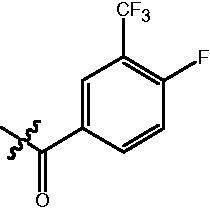	20.29 ± 0.29	17.37 ± 3.35
SAHA		58.27 ± 6.06	96.93 ± 0.17

^a^Illustrated as percentage inhibitory rate, and each value is the mean of three experiments.

**Table 2.** Enzyme inhibitory and antiproliferative activities of Fb series compounds (percentage of inhibition at concentration of 1 µM). 
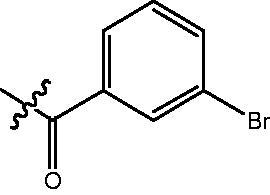

Compounds	R	HDACs^a^	U937^a^
Fb-1	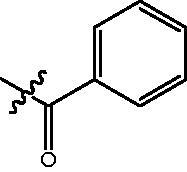	57.50 ± 2.92	65.20 ± 0.29
Fb-2	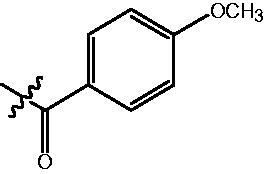	52.30 ± 2.26	94.46 ± 0.41
Fb-3	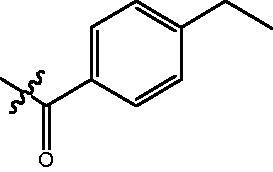	53.90 ± 1.82	96.05 ± 0.01
Fb-4	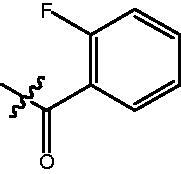	66.54 ± 1.47	95.88 ± 1.09
Fa-5	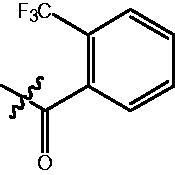	65.08 ± 11.09	33.95 ± 1.70
Fb-6	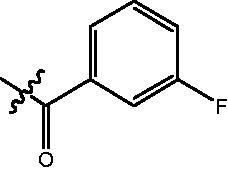	58.36 ± 5.06	92.19 ± 2.72
Fb-7	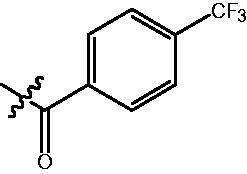	47.03 ± 15.05	84.91 ± 1.68
Fb-8	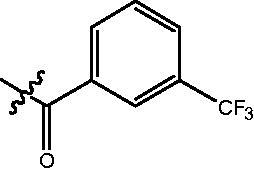	47.09 ± 0.62	78.16 ± 3.05
Fb-9	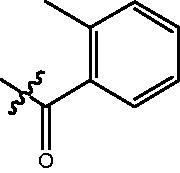	35.97 ± 3.07	69.20 ± 3.08
Fb-10	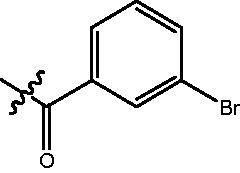	49.47 ± 9.25	86.20 ± 4.18
Fb-11	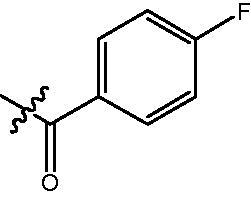	48.27 ± 0.62	59.18 ± 6.95
Fb-12	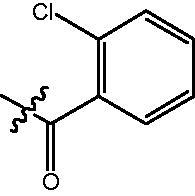	59.61 ± 6.64	76.64 ± 6.97
Fb-13	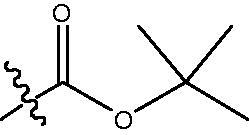	48.09 ± 3.01	75.50 ± 2.40
Fb-14	H	58.84 ± 2.40	75.63 ± 2.28
SAHA		56.23 ± 2.12	88.15 ± 1.62

^a^Illustrated as percentage inhibitory rate, and each value is the mean of three experiments.

Different subtypes of HDACs may play different biological effects in cells. Therefore, it is important to determine the inhibitory selectivity of active HDAC inhibitors. In the present study, the selectivity of representative compound **Fb-4** was tested against HDAC1, 2, 3, 6 and 8 (Table 3). The results revealed that compound **Fb-4** has no obvious HDAC isoform selectivity in the inhibition of HDAC1 (IC_50_ value of 12.1 nM), HDAC2 (IC_50_ value of 21.5 nM), HDAC3 (IC_50_ value of 11.0 nM) and HDAC6 (IC_50_ value of 8.6 nM). Whereas, in the inhibition of HDAC8, compound **Fb-4** exhibited reduced potency with IC_50_ value of 304 nM. It is indicated that molecule **Fb-4** is a pan HDAC inhibitor.

**Table 3.** HDAC isoform selectivity of Fb-4 compared with SAHA (IC_50_, nM^a^)

Cell line	Fb-4	SAHA
HDAC1	12.1 ± 2.5	46.6 ± 16.4
HDAC2	21.5 ± 4.5	165.3 ± 1.1
HDAC3	11.0 ± 2.8	27.2 ± 4.4
HDAC6	8.6 ± 1.3	21.8 ± 2.2
HDAC8	304.0 ± 32.6	>5000

^a^Each value is the mean of three experiments.

### Antiproliferative activity

All the synthesised molecules were screened against U937 cells (Tables 1 and 2), and only active molecules which exhibited good performances in both enzymatic and antiproliferative test were selected for the cancer cell growth inhibitory assay against various cell lines. In the U937 cell based screening, The **Fb** series molecules with mean inhibitory rate of 77.32 also showed significantly improved activities compared with the **Fa** series molecules (with mean inhibitory rate of 20.82). Obviously, compound **Fb-2**, **Fb-3** and **Fb-4** displayed inhibitory rate of 94.46%, 96.05% and 95.88% compared with SAHA (inhibitory rate of 96.93%). Therefore, IC_50_ values of **Fb-2**, **Fb-3** and **Fb-4** against different kinds of cancer cells were calculated.

K562, U266, MCF-7, U937 and HEPG2 cell lines were used in the further antiproliferative test. In the inhibition of K562, U266, MCF-7 and HEPG2 cells, all the tested compounds showed improved activities compared with SAHA (Table 4). The IC_50_ values of **Fb-2**, **Fb-3** and **Fb-4** against K562 cells were 1.19, 0.47 and 0.87 µM, respectively (IC_50_ values of SAHA was 1.34 µM). U266 cells were sensitive to the tested molecules, and IC_50_ values of **Fb-2**, **Fb-3** and **Fb-4** against U266 cells were 0.09, 0.10 and 0.09 µM, respectively, compared with SAHA (0.14 µM). Compared with SAHA (IC_50_ value of 2.58 µM), the tested molecules exhibited obviously increased activities in the inhibition of MCF-7 cells, especially **Fb-4** with IC_50_ value of 0.32 µM. In the U937 cell based assay, **Fb-3** (IC_50_ value of 0.63 µM) and **Fb-4** (IC_50_ value of 0.34 µM) showed improved inhibitory activity compared with SAHA (IC_50_ value of 0.67 µM). HEPG2 cells were not as sensitive as other tested cell lines, and the tested compound showed IC_50_ values of 17.86 (**Fb-2**), 14.86 (**Fb-3**) and 17.37 (**Fb-4**) µM respectively, compared with SAHA (IC_50_ value of 18.17 µM). Collectively, compound **Fb-4** exhibited the best performance in the *in vitro* antiproliferative test. Therefore, **Fb-4** was selected for further cell cycle and apoptosis analysis.

**Table 4.** Antiproliferative activities of Fb-2, Fb-3 and Fb-4 against various cancer cell lines (IC_50_, μM^a^)

Cell line	Tumour type	Fb-2	Fb-3	Fb-4	SAHA
K562	Leukemia	1.19 ± 0.06	0.47 ± 0.03	0.87 ± 0.01	1.34 ± 0.04
U266	Multiple myeloma	0.09 ± 0.01	0.10 ± 0.02	0.09 ± 0.01	0.14 ± 0.06
MCF-7	Breast carcinoma	0.94 ± 0.02	0.53 ± 0.09	0.32 ± 0.04	2.58 ± 0.14
U937	Lymphoma	0.92 ± 0.09	0.63 ± 0.02	0.34 ± 0.06	0.67 ± 0.06
HEPG2	Liver cancer	17.86 ± 1.04	14.86 ± 0.78	17.37 ± 0.63	18.17 ± 0.92

^a^Each value is the mean of three experiments.

### Cell cycle analysis

**Figure 2.**
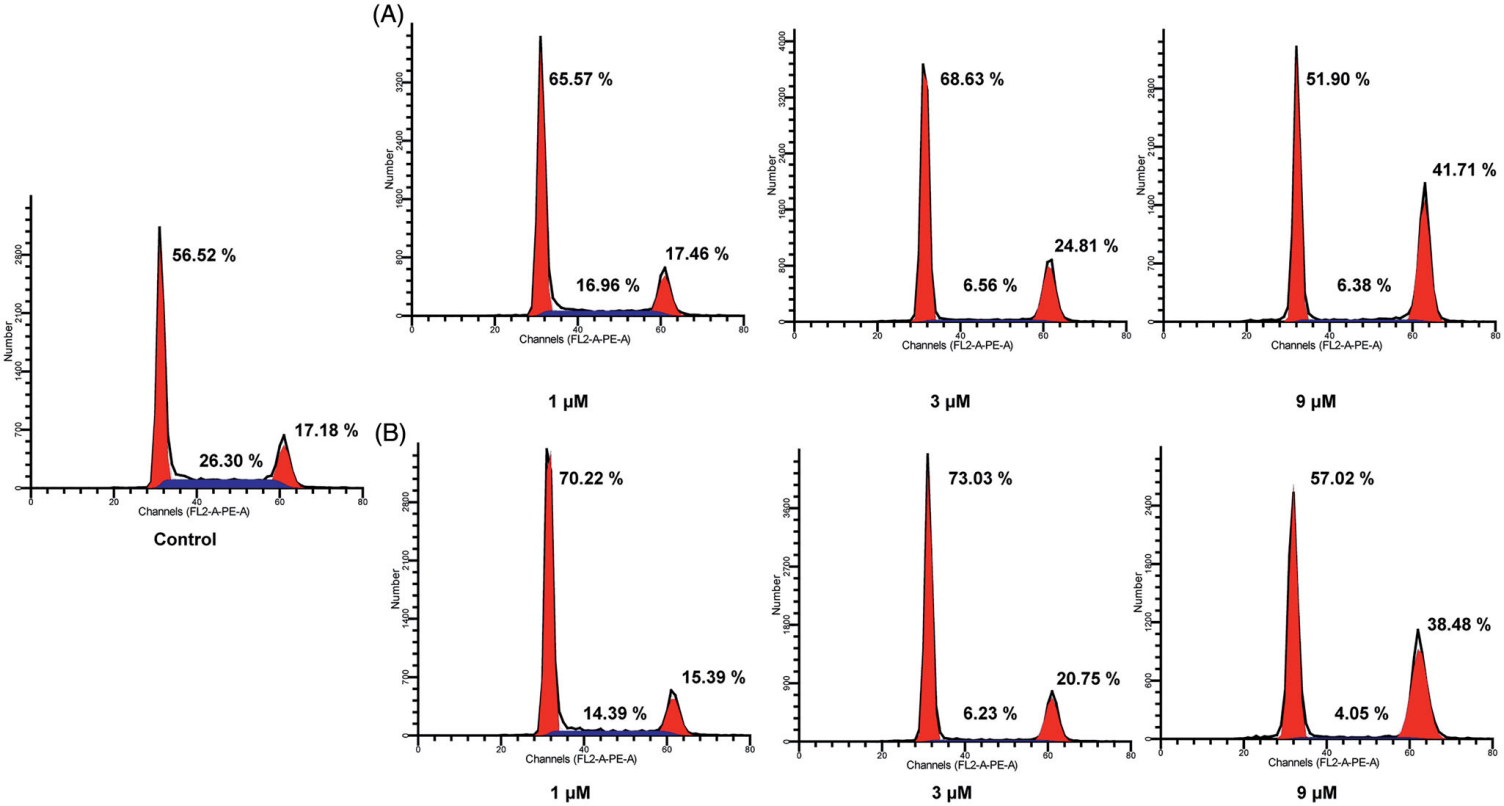
Cell cycle analysis of molecule **Fb-4** on MCF-7cells. Cells were treated with **Fb-4** (A) and SAHA (B) at the concentration of 1, 3 and 9 µM for 24 h. The results were detected by flow cytometry analysis.

The cell cycle is divided into three distinct phases including G0/G1 phase, S phase, and G2/M phase. G2/M is important for the entrance of cells into the M phase. To verify the causal relation of cell proliferation inhibition of **Fb-4** and cell cycle arrest, the cell cycle distribution was analysed by treating MCF-7 cells with various doses of **Fb-4** and SAHA (1, 3 and 9 µM) for 24 h. As shown in Figure 2, both **Fb-4** (Figure 2(A)) and SAHA (Figure 2(B)) increased cell number at G2/M phase with raising concentration, accompanied by decreased cell number at S phase in MCF-7 cells. Compared with SAHA (15.39%, 20.75% and 38.48% at the concentration of 1, 3 and 9 µM, respectively), molecule **Fb-4** exhibited significant effects in the G2/M phase arrest of MCF-7 cells with cell percentage of 17.46, 24.81, and 41.71 at the concentration of 1, 3 and 9 µM, respectively. It is demonstrated that treatment of MCF-7 cells with **Fb-4** could effectively inhibit cell proliferation via induction of cell cycle arrest at G2/M phase. It is suggested that molecule **Fb-4** could inhibit cell proliferation of MCF-7 cells by inhibiting protein synthesis and rapid cell growth as a result of G2/M phase arrest.

### Apoptotic analysis

**Figure 3.**
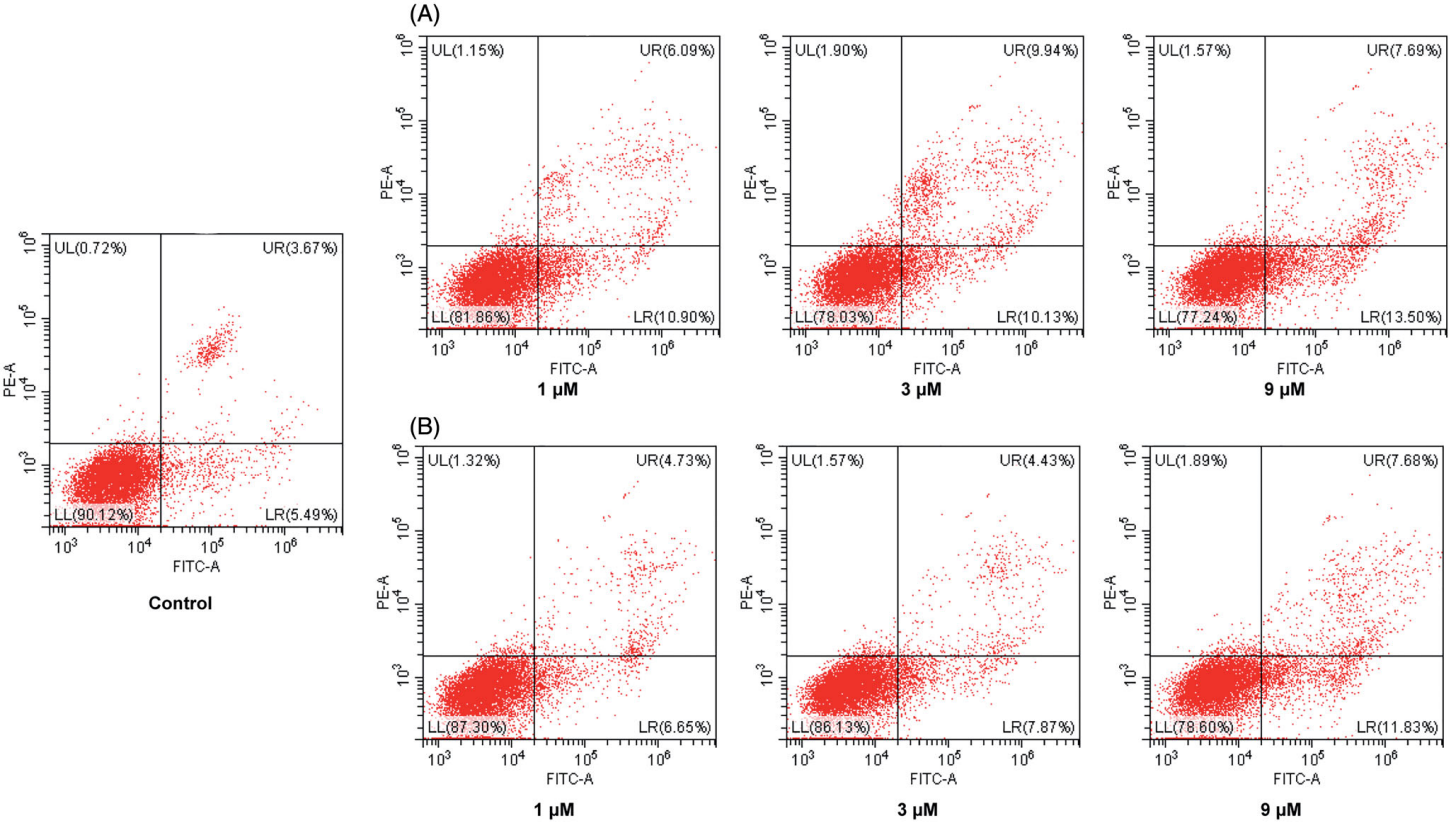
Pro-apoptotic effect of molecule **Fb-4**. MCF-7cells were treated with **Fb-4** (A) and SAHA (B) at the concentration of 1, 3 and 9 µM for 24 h. Then cells were stained with Annexin V-FITC/PI, and the results were detected by flow cytometry analysis.

Apoptosis plays an important role in the treatment of cancer. Moreover, efforts to increase G2/M arrest have also been associated with enhanced apoptosis. Therefore, to determine whether the cell cycle arrest caused by molecule **Fb-4** can ultimately induce apoptosis, MCF-7 cells were treated with different concentrations of molecule **Fb-4** (1, 3 and 9 µM). As shown in Figure 3, increased number of apoptotic cells was detected in MCF-7 cells treated with various doses of **Fb-4** (3 A) and SAHA (3B). It is significant that both **Fb-4** and SAHA induced MCF-7 cell apoptosis in a dose dependent manner. After treatment with difference concentrations of **Fb-4** (1, 3 and 9 µM), the percentage of apoptotic cells were significantly increased from 9.16% of the control to 16.99%, 20.07% and 21.19%, respectively, compared with SAHA (11.38%, 12.30% and 19.51% at the concentration of 1, 3 and 9 µM, respectively). It is indicated that induction of cell apoptosis makes contributions to the anti-proliferative effect of compound **Fb-4**.

## Conclusion

In discovery of lead compound for the anticancer drug development, pharmacophores of phenanthridine structure were introduced to the cap region of HDAC inhibitors. Two series of novel HDAC inhibitors were synthesised with different linkers. In the enzyme inhibitory and *in vitro* antiproliferative screening, target compounds with aromatic linker exhibited improved activities compared with molecules with fatty linker. In the *in vitro* cancer cell based test, the selected compounds showed potency in the inhibition of both solid (MCF-7 and HEPG2 cells) and haematologic (K562, U266 and U937 cells) tumour cell lines compared with SAHA. Significantly, compared with SAHA, molecule **Fb-4** displayed 0.87, 0.09, 0.32, 0.34 and 17.37 µM of IC_50_ values against K562, U266, MCF-7, U937 and HEPG2 cells, respectively. Cell cycle and apoptotic analysis revealed that induction of G2/M phase arrest and apoptosis relate to the antiproliferative potency of **Fb-4**. Collectively, a potent lead compound (**Fb-4**) was discovered for the treatment of cancer by inhibition of HDACs. It must be pointed out that molecules with aromatic linker have poor solubility in both aqueous and lipid solutions. Therefore, structural modification of compound **Fb-4** will be performed by improving the pharmacokinetic profiles and anticancer potency in our further research.

## Materials and methods

All chemicals were obtained from commercial suppliers and can be used without further refinement. All reactions were detected by TLC using 0.25 mm silica gel plate (60GF-254). UV light and ferric chloride were used to show TLC spots. Due to the poor solubility of the target compounds, only the ^1^H NMR spectra were derived for the structural identification. ^1^H NMR spectra were recorded on a Bruker DRX spectrometer at 500 MHz, using TMS as an internal standard.

Compound 1–3 were synthesised as described in our previous work^13^.

### Synthesis of compound 4

4–(6-tert-Butoxycarbonylamino-benzo[1,3]dioxol-5-yl)-benzoic acid methyl ester. Compound **3** was dissolved (0.5 g, 1.59 mmol) in a mixed solution of 1,4-dioxane and water (20:1, 21 ml), K_2_CO_3_ (0.44 g, 3.18 mmol) and Trans-Dichlorobis (triphenyl-phosphine)Palladium(II) (0.11 g, 0.16 mmol) were added, and refluxed for 12 h under argon protection. After the reaction, the reagents were spin-dried under vacuum. The mixture was dissolved by EtOAc (100 ml), washed with saturated NaHCO_3_ (3 × 20 ml) and brine (1 × 20 ml), dried over MgSO_4_, and evaporated under vacuum. The crude product was purified by silica gel column chromatography to obtain compound **4** (0.34 g, 58% yield) as a pale yellow oil. ^1^H NMR (400 MHz, DMSO) δ 8.43 (s, 1H), 7.96 (d, *J* = 8.2 Hz, 2H), 7.48 (d, *J* = 8.1 Hz, 2H), 6.91 (s, 1H), 6.85 (s, 1H), 6.08 (s, 2H), 3.87 (s, 3H), 1.26 (s, 9H).

### Synthesis of compound 5

4–(6-tert-Butoxycarbonylamino-benzo[1,3]dioxol-5-yl)-benzoic acid. Compound 4 (4.0 g, 10.78 mmol) was added to methanol and 3 mol/l NaOH (1:1,80 ml), and reacted at 40 °C for about 10 h. After the reaction is over, it was evaporated to dryness by rotary evaporation, added appropriate amount of water, adjusted the PH to acidity with 3 mol/l HCL, added EtOAc for extraction (3 × 100 ml), and washed the combined EtOAc layer once with brine, dried over MgSO_4_, and evaporated under vacuo. The desired compound **5** (3.43 g, 89% yield) was derived by crystallisation in EtOAc as white powder.^1^H NMR (400 MHz, DMSO) δ 12.91 (s, 1H), 8.40 (s, 1H), 7.93 (d, *J* = 8.2 Hz, 2H), 7.44 (d, *J* = 8.0 Hz, 2H), 6.91 (s, 1H), 6.85 (s, 1H), 6.07 (s, 2H), 1.25 (s, 9H).

### Synthesis of compound 6a

[4-(6-tert-Butoxycarbonylamino-benzo[1,3]dioxol-5-yl)-benzoylamino]-butyric acid methyl ester **(6a)**. To a solution of compound **5** (2.0 g, 8.47 mmol) in DCM (50 mL), Et_3_N (1.03 g, 10.17 mmol) and TBTU (3.26 g, 10.17 mmol) were sequentially added. After 20 min, methyl 4-aminobutyrate hydrochloride (1.07 g, 10.17 mmol) and Et_3_N (1.03 g, 10.17 mmol) were added. The reaction was stirred at room temperature for 5h. After the reaction, the solvent was evaporated in vacuo, and the residue was taken up in EtOAc (50 mL). The EtOAc solution was washed with saturated citric acid (3 × 20 mL), NaHCO_3_ (3 × 20 mL) and brine (3 × 20 mL), dried over MgSO_4_ and concentrated by evaporation in vacuo. The desired compound **6a** (3.28 g, 85% yield) was derived by crystallisation in EtOAc as white powder. ^1^H NMR (400 MHz, DMSO) δ 8.49 (t, *J* = 5.5 Hz, 1H), 8.33 (s, 1H), 7.86 (d, *J* = 8.2 Hz, 2H), 7.40 (d, *J* = 8.1 Hz, 2H), 6.89 (s, 1H), 6.86 (s, 1H), 6.07 (s, 2H), 3.59 (s, 3H), 3.29 (dd, *J* = 12.8, 6.8 Hz, 2H), 2.37 (t, *J* = 7.4 Hz, 2H), 1.79 (p, *J* = 7.1 Hz, 2H), 1.27 (s,9H).

### Intermediate 6b was prepared as described for 6a

4-{[4–(6-tert-Butoxycarbonylamino-benzo[1,3]dioxol-5-yl)-benzoylamino]-methyl}-benzoic acid methyl ester **(6b)**. ^1^H NMR (400 MHz, DMSO) δ 9.17 (t, *J* = 5.9 Hz, 1H), 8.36 (s, 1H), 7.94 (d, *J* = 8.2 Hz, 4H), 7.44 (dd, *J* = 8.0, 4.0 Hz, 4H), 6.90 (s, 1H), 6.87 (s, 1H), 6.07 (s, 2H), 4.58 (d, *J* = 5.8 Hz, 2H), 3.84 (s, 3H), 1.28 (s, 9H).

### Preparation of fa-1 and its analogues: Derivatives fa-fb were prepared as described for fa-1 (see below)

Compound **6a** (0.4 g, 0.88 mmol) was dissolved in dry DCM. After addition of TFA, the solvent was stirred at room temperature, monitored by TLC. Until the raw materials were completely consumed, the reagents were evaporated under vacuum and dissolved with a mixed solution of tetrahydrofuran and water (50:1) at 0 °C with addition of sodium bicarbonate. After stirred for 10 min, 3-Bromobenzoyl chloride (0.23 g, 1.03 mmol) was added, and the misture was stirred at room temperature for 4 h. After that, the reagents were evaporated under vacuum and dissolved in EtOAc. The solvent was washed with saturated NaHCO_3_ (3 × 20 ml) and brine (1 × 20 ml), dried over MgSO_4_, and evaporated under vacuo. The desired compound **fa-1** (0.43 g, 91% yield) was derived by crystallisation in EtOAc as white powder.

4-{4-[6-(3-Bromo-benzoylamino)-benzo[1,3]dioxol-5-yl]-benzoylamino}-butyric acid methyl ester **(fa-1)**. ^1^H NMR (400 MHz, DMSO) δ 9.95 (s, 1H), 8.46 (t, *J* = 5.5 Hz, 1H), 7.94 (s, 1H), 7.84 − 7.71 (m, 4H), 7.43 (dd, *J* = 14.8, 8.0 Hz, 3H), 7.01 (d, *J* = 11.0 Hz, 2H), 6.12 (s, 2H), 3.55 (s, 3H), 3.26 (dd, *J* = 12.6, 6.7 Hz, 2H), 2.35 (t, *J* = 7.4 Hz, 2H), 1.76 (p, *J* = 7.1 Hz, 2H).

### Intermediate fa-2 through fa-23, fb-1 through fb-14 were prepared as described for fa-1

4-{4-[6-(2-Methoxy-benzoylamino)-benzo[1,3]dioxol-5-yl]-benzoylamino}-butyric acid methyl ester **(fa-2)**. ^1^H NMR (400 MHz, DMSO) δ 9.76 (s, 1H), 8.56 (t, *J* = 5.5 Hz, 1H), 7.92 (dd, *J* = 10.8, 4.9 Hz, 3H), 7.81 (s, 1H), 7.51 (d, *J* = 8.3 Hz, 3H), 7.07 (t, *J* = 7.4 Hz, 2H), 6.90 (s, 1H), 6.10 (s, 2H), 3.59 (s, 3H), 3.39 (s, 3H), 3.32 − 3.26 (m, 2H), 2.38 (t, *J* = 7.4 Hz, 2H), 1.80 (p, *J* = 7.1 Hz, 2H).

4-{4-[6-(4-Fluoro-benzoylamino)-benzo[1,3]dioxol-5-yl]-benzoylamino}-butyric acid methyl ester **(fa-3)**. ^1^H NMR (400 MHz, DMSO) δ 9.86 (s, 1H), 8.47 (t, *J* = 5.4 Hz, 1H), 7.85 (dd, *J* = 8.5, 5.6 Hz, 2H), 7.80 (d, *J* = 8.4 Hz, 2H), 7.46 (d, *J* = 8.3 Hz, 2H), 7.29 (t, *J* = 8.8 Hz, 2H), 7.00 (d, *J* = 8.7 Hz, 2H), 6.12 (s, 2H), 3.55 (s, 3H), 3.25 (dd, *J* = 12.7, 6.7 Hz, 2H), 2.35 (t, *J* = 7.4 Hz, 2H), 1.76 (p, *J* = 7.2 Hz, 2H).

4-{4-[6-(2-Methyl-benzoylamino)-benzo[1,3]dioxol-5-yl]-benzoylamino}-butyric acid methyl ester **(fa-4)**. ^1^H NMR (400 MHz, DMSO) δ 9.68 (s, 1H), 8.51 (t, *J* = 5.4 Hz, 1H), 7.86 (d, *J* = 8.2 Hz, 2H), 7.47 (d, *J* = 8.1 Hz, 2H), 7.31 − 7.23 (m, 2H), 7.21 (d, *J* = 6.7 Hz, 2H), 7.04 (s, 1H), 6.96 (s, 1H), 6.11 (s, 2H), 3.58 (s, 3H), 3.30 − 3.24 (m, 2H), 2.36 (t, *J* = 7.4 Hz, 2H), 2.20 (s, 3H), 1.85 − 1.73 (m, 2H).

4-{4-[6-(3-Fluoro-benzoylamino)-benzo[1,3]dioxol-5-yl]-benzoylamino}-butyric acid methyl ester **(fa-5)**. ^1^H NMR (400 MHz, DMSO) δ 10.07 (s, 1H), 8.43 (t, *J* = 5.3 Hz, 1H), 8.07 (d, *J* = 9.1 Hz, 2H), 7.91 (d, *J* = 7.7 Hz, 1H), 7.80 (d, *J* = 8.5 Hz, 2H), 7.71 (t, *J* = 7.7 Hz, 1H), 7.49 − 7.44 (m, 2H), 7.05 (s, 1H), 7.01 (t, *J* = 5.1 Hz, 1H), 6.13 (d, *J* = 2.0 Hz, 2H), 3.55 (d, *J* = 1.6 Hz, 3H), 3.25 (dd, *J* = 12.7, 6.6 Hz, 2H), 2.34 (t, *J* = 7.4 Hz, 2H), 1.76 (p, *J* = 7.1 Hz, 2H).

4-{4-[6-(2-Trifluoromethyl-benzoylamino)-benzo[1,3]dioxol-5-yl]-benzoylamino}-butyric acid methyl ester **(fa-6)**. ^1^H NMR (400 MHz, DMSO) δ 10.00 (s, 1H), 8.55 (s, 1H), 7.90 (d, *J* = 8.2 Hz, 2H), 7.78 (d, *J* = 7.7 Hz, 1H), 7.72 (t, *J* = 7.4 Hz, 1H), 7.64 (t, *J* = 7.6 Hz, 1H), 7.48 (d, *J* = 8.4 Hz, 2H), 7.40 (d, *J* = 7.5 Hz, 1H), 6.98 (d, *J* = 2.6 Hz, 2H), 6.12 (s, 2H), 3.58 (d, *J* = 4.3 Hz, 3H), 3.31 − 3.25 (m, 2H), 2.37 (t, *J* = 7.4 Hz, 2H), 1.79 (p, *J* = 7.2 Hz, 2H).

4-{4-[6-(4-Ethyl-benzoylamino)-benzo[1,3]dioxol-5-yl]-benzoylamino}-butyric acid methyl ester **(fa-7)**. ^1^H NMR (400 MHz, DMSO) δ 9.73 (s, 1H), 8.47 (t, *J* = 5.4 Hz, 1H), 7.80 (d, *J* = 8.4 Hz, 2H), 7.70 (d, *J* = 8.1 Hz, 2H), 7.46 (d, *J* = 8.4 Hz, 2H), 7.27 (d, *J* = 8.2 Hz, 2H), 7.00 (d, *J* = 10.1 Hz, 2H), 6.12 (s, 2H), 3.55 (s, 3H), 3.25 (dd, *J* = 12.6, 6.7 Hz, 2H), 2.63 (q, *J* = 7.5 Hz, 2H), 2.34 (t, *J* = 7.4 Hz, 2H), 1.76 (p, *J* = 7.2 Hz, 2H), 1.18 (t, *J* = 7.5 Hz, 3H).

4-{4-[6-(2-Fluoro-benzoylamino)-benzo[1,3]dioxol-5-yl]-benzoylamino}-butyric acid methyl ester **(fa-8)**. ^1^H NMR (400 MHz, DMSO) δ 9.72 (d, *J* = 2.0 Hz, 1H), 8.50 (t, *J* = 5.5 Hz, 1H), 7.86 (d, *J* = 8.4 Hz, 2H), 7.56 − 7.45 (m, 4H), 7.26 (dd, *J* = 13.3, 5.7 Hz, 2H), 7.12 (s, 1H), 6.97 (s, 1H), 6.12 (s, 2H), 3.57 (s, 3H), 3.28 (dd, *J* = 12.7, 6.7 Hz, 2H), 2.36 (t, *J* = 7.4 Hz, 2H), 1.78 (p, *J* = 7.2 Hz, 2H).

4-{4-[6-(3-Trifluoromethyl-benzoylamino)-benzo[1,3]dioxol-5-yl]-benzoylamino}-butyric acid methyl ester **(fa-9)**. ^1^H NMR (400 MHz, DMSO) δ 10.07 (s, 1H), 8.44 (t, *J* = 5.6 Hz, 1H), 8.07 (d, *J* = 9.0 Hz, 2H), 7.91 (d, *J* = 7.8 Hz, 1H), 7.80 (d, *J* = 8.5 Hz, 2H), 7.71 (t, *J* = 7.7 Hz, 1H), 7.47 (d, *J* = 8.3 Hz, 2H), 7.05 (s, 1H), 7.01 (s, 1H), 6.13 (s, 2H), 3.55 (s, 3H), 3.25 (dd, *J* = 12.6, 6.7 Hz, 2H), 2.34 (t, *J* = 7.4 Hz, 2H), 1.76 (p, *J* = 7.2 Hz, 2H).

4-{4-[6-(2-Chloro-benzoylamino)-benzo[1,3]dioxol-5-yl]-benzoylamino}-butyric acid methyl ester **(fa-10)**. ^1^H NMR (400 MHz, DMSO) δ 9.89 (s, 1H), 8.51 (t, *J* = 5.6 Hz, 1H), 7.87 (d, *J* = 8.4 Hz, 2H), 7.52 − 7.46 (m, 3H), 7.39 (dddd, *J* = 17.7, 9.6, 7.2, 2.1 Hz, 3H), 7.06 (s, 1H), 6.97 (s, 1H), 6.12 (s, 2H), 3.58 (s, 3H), 3.29 (dd, *J* = 12.7, 6.7 Hz, 2H), 2.37 (t, *J* = 7.4 Hz, 2H), 1.79 (p, *J* = 7.2 Hz, 2H).

4-[4–(6-Benzoylamino-benzo[1,3]dioxol-5-yl)-benzoylamino]-butyric acid methyl ester **(fa-11)**. ^1^H NMR (400 MHz, DMSO) δ 9.80 (s, 1H), 8.44 (t, *J* = 5.6 Hz, 1H), 7.78 (dd, *J* = 10.5, 8.3 Hz, 4H), 7.56 − 7.50 (m, 1H), 7.49 − 7.42 (m, 4H), 7.02 (s, 1H), 6.99 (s, 1H), 6.12 (s, 2H), 3.55 (s, 3H), 3.25 (dd, *J* = 12.6, 6.7 Hz, 2H), 2.34 (t, *J* = 7.4 Hz, 2H), 1.76 (p, *J* = 7.2 Hz, 2H).

4-{4-[6-(4-Trifluoromethyl-benzoylamino)-benzo[1,3]dioxol-5-yl]-benzoylamino}-butyric acid methyl ester **(fa-12)**. ^1^H NMR (400 MHz, DMSO) δ 10.06 (s, 1H), 8.44 (t, *J* = 5.6 Hz, 1H), 7.96 (d, *J* = 8.1 Hz, 2H), 7.82 (dd, *J* = 18.1, 8.4 Hz, 4H), 7.46 (d, *J* = 8.3 Hz, 2H), 7.05 (s, 1H), 7.01 (s, 1H), 6.13 (s, 2H), 3.55 (s, 3H), 3.25 (dd, *J* = 12.6, 6.7 Hz, 2H), 2.34 (t, *J* = 7.4 Hz, 2H), 1.76 (p, *J* = 7.2 Hz, 2H).

4-{4-[6-(4-Methoxy-benzoylamino)-benzo[1,3]dioxol-5-yl]-benzoylamino}-butyric acid methyl ester **(fa-13)**. ^1^H NMR (400 MHz, DMSO) δ 9.64 (s, 1H), 8.45 (t, *J* = 5.5 Hz, 1H), 7.77 (t, *J* = 9.2 Hz, 4H), 7.45 (d, *J* = 8.0 Hz, 2H), 6.98 (t, *J* = 8.7 Hz, 4H), 6.11 (s, 2H), 3.79 (s, 3H), 3.55 (s, 3H), 3.25 (q, *J* = 6.5 Hz, 2H), 2.34 (t, *J* = 7.4 Hz, 2H), 1.76 (p, *J* = 7.1 Hz, 2H).

4-{4-[6–(3,5-Difluoro-benzoylamino)-benzo[1,3]dioxol-5-yl]-benzoylamino}-butyric acid methyl ester **(fa-14)**. ^1^H NMR (400 MHz, DMSO) δ 9.83 (s, 1H), 8.50 (t, *J* = 5.5 Hz, 1H), 7.86 (d, *J* = 8.1 Hz, 2H), 7.48 (d, *J* = 8.1 Hz, 2H), 7.41 − 7.28 (m, 3H), 7.12 (s, 1H), 6.98 (s, 1H), 6.12 (s, 2H), 3.57 (s, 3H), 3.28 (dd, *J* = 12.7, 6.5 Hz, 2H), 2.36 (t, *J* = 7.4 Hz, 2H), 1.78 (p, *J* = 7.2 Hz, 2H).

4-{4-[6–(2,4-Difluoro-benzoylamino)-benzo[1,3]dioxol-5-yl]-benzoylamino}-butyric acid methyl ester **(fa-15)**. ^1^H NMR (400 MHz, DMSO) δ 9.73 (s, 1H), 8.49 (t, *J* = 5.5 Hz, 1H), 7.85 (d, *J* = 8.1 Hz, 2H), 7.60 (dd, *J* = 15.4, 8.2 Hz, 1H), 7.48 (d, *J* = 8.1 Hz, 2H), 7.37 − 7.29 (m, 1H), 7.17 (t, *J* = 8.5 Hz, 1H), 7.12 (s, 1H), 6.97 (s, 1H), 6.12 (s, 2H), 3.57 (s, 3H), 3.28 (dd, *J* = 12.7, 6.5 Hz, 2H), 2.37 (t, *J* = 7.4 Hz, 2H), 1.78 (p, *J* = 7.2 Hz, 2H).

4-{4-[6–(4-Trifluoromethoxy-benzoylamino)-benzo[1,3]dioxol-5-yl]-benzoylamino}-butyric acid methyl ester **(fa-16)**. ^1^H NMR (400 MHz, DMSO) δ 9.94 (s, 1H), 8.44 (t, *J* = 5.5 Hz, 1H), 7.89 (d, *J* = 8.5 Hz, 2H), 7.80 (d, *J* = 8.2 Hz, 2H), 7.46 (d, *J* = 8.0 Hz, 4H), 7.01 (d, *J* = 11.2 Hz, 2H), 6.12 (s, 2H), 3.55 (s, 3H), 3.25 (dd, *J* = 12.7, 6.5 Hz, 2H), 2.35 (t, *J* = 7.4 Hz, 2H), 1.76 (p, *J* = 7.1 Hz, 2H).

4-{4-[6-(3-Nitro-benzoylamino)-benzo[1,3]dioxol-5-yl]-benzoylamino}-butyric acid methyl ester **(fa-17)**. ^1^H NMR (400 MHz, DMSO) δ 10.21 (s, 1H), 8.60 (s, 1H), 8.44 (t, *J* = 5.5 Hz, 1H), 8.38 (d, *J* = 8.3 Hz, 1H), 8.21 (d, *J* = 7.8 Hz, 1H), 7.78 (dd, *J* = 15.4, 8.0 Hz, 3H), 7.47 (d, *J* = 8.1 Hz, 2H), 7.04 (d, *J* = 18.0 Hz, 2H), 6.13 (s, 2H), 3.55 (s, 3H), 3.24 (q, *J* = 6.5 Hz, 2H), 2.34 (t, *J* = 7.4 Hz, 2H), 1.75 (p, *J* = 7.1 Hz, 2H).

### 4-{4-[6-(4-Fluoro-2-trifluoromethyl-benzoylamino)-benzo[1,3]dioxol-5-yl]-benzoylamino}-butyric acid methyl ester (fa-18)

^1^H NMR (400 MHz, DMSO) δ 9.99 (s, 1H), 8.45 (t, *J* = 5.5 Hz, 1H), 7.81 (d, *J* = 8.2 Hz, 2H), 7.46 (dd, *J* = 12.6, 7.5 Hz, 6H), 7.01 (d, *J* = 7.7 Hz, 2H), 6.13 (s, 2H), 3.55 (s, 3H), 3.26 (q, *J* = 6.5 Hz, 2H), 2.35 (t, *J* = 7.4 Hz, 2H), 1.76 (p, *J* = 7.1 Hz, 2H).

4-{4-[6-(3-Chloro-4-fluoro-benzoylamino)-benzo[1,3]dioxol-5-yl]-benzoylamino}-butyric acid methyl ester **(fa-19)**. ^1^H NMR (400 MHz, DMSO) δ 10.01 (s, 1H), 8.51 (t, *J* = 5.5 Hz, 1H), 7.88 (d, *J* = 8.1 Hz, 2H), 7.73 (dd, *J* = 9.3, 2.2 Hz, 1H), 7.62 (dd, *J* = 9.5, 7.4 Hz, 1H), 7.48 (t, *J* = 8.4 Hz, 3H), 6.98 (d, *J* = 5.4 Hz, 2H), 6.12 (s, 2H), 3.58 (s, 3H), 3.29 (q, *J* = 6.6 Hz, 2H), 2.37 (t, *J* = 7.4 Hz, 2H), 1.79 (p, *J* = 7.1 Hz, 2H).

4-{4-[6-(4-Propyl-benzoylamino)-benzo[1,3]dioxol-5-yl]-benzoylamino}-butyric acid methyl ester **(fa-20)**. ^1^H NMR (400 MHz, DMSO) δ 9.72 (s, 1H), 8.45 (t, *J* = 5.5 Hz, 1H), 7.80 (d, *J* = 8.2 Hz, 2H), 7.69 (d, *J* = 7.9 Hz, 2H), 7.46 (d, *J* = 8.1 Hz, 2H), 7.26 (d, *J* = 8.0 Hz, 2H), 7.00 (d, *J* = 10.9 Hz, 2H), 6.11 (s, 2H), 3.55 (s, 3H), 3.25 (q, *J* = 6.5 Hz, 2H), 2.58 (t, *J* = 7.5 Hz, 2H), 2.34 (t, *J* = 7.4 Hz, 2H), 1.76 (p, *J* = 7.1 Hz, 2H), 1.64 − 1.53 (m, 2H), 0.88 (t, *J* = 7.3 Hz, 3H).

4-{4-[6–(2-Chloro-6-fluoro-benzoylamino)-benzo[1,3]dioxol-5-yl]-benzoylamino}-butyric acid methyl ester **(fa-21)**. ^1^H NMR (400 MHz, DMSO) δ 9.95 (s, 1H), 8.45 (t, *J* = 5.5 Hz, 1H), 7.98 (d, *J* = 7.6 Hz, 1H), 7.80 (d, *J* = 8.1 Hz, 3H), 7.52 (t, *J* = 8.9 Hz, 1H), 7.45 (d, *J* = 8.1 Hz, 2H), 7.01 (d, *J* = 7.9 Hz, 2H), 6.12 (s, 2H), 3.56 (s, 3H), 3.25 (dd, *J* = 12.7, 6.5 Hz, 2H), 2.35 (t, *J* = 7.4 Hz, 2H), 1.76 (p, *J* = 7.1 Hz, 2H).

4-{4-[6-(2,5-Difluoro-benzoylamino)-benzo[1,3]dioxol-5-yl]-benzoylamino}-butyric acid methyl ester **(fa-22)**. ^1^H NMR (400 MHz, DMSO) δ 10.16 (s, 1H), 8.51 (t, *J* = 5.5 Hz, 1H), 7.87 (d, *J* = 8.1 Hz, 2H), 7.47 (dd, *J* = 13.5, 8.1 Hz, 3H), 7.36 (d, *J* = 8.1 Hz, 1H), 7.29 (t, *J* = 8.6 Hz, 1H), 7.01 (d, *J* = 23.4 Hz, 2H), 6.13 (s, 2H), 3.59 (s, 3H), 3.29 (dd, *J* = 12.8, 6.6 Hz, 2H), 2.37 (t, *J* = 7.4 Hz, 2H), 1.79 (p, *J* = 7.1 Hz, 2H).

4-{4-[6-(4-Fluoro-3-trifluoromethyl-benzoylamino)-benzo[1,3]dioxol-5-yl]-benzoylamino}-butyric acid methyl ester **(fa-23)**. ^1^H NMR (400 MHz, DMSO) δ 10.09 (s, 1H), 8.44 (t, *J* = 5.5 Hz, 1H), 8.14 (d, *J* = 6.6 Hz, 2H), 7.80 (d, *J* = 8.2 Hz, 2H), 7.64 (t, *J* = 9.9 Hz, 1H), 7.46 (d, *J* = 8.1 Hz, 2H), 7.03 (d, *J* = 17.9 Hz, 2H), 6.13 (s, 2H), 3.55 (s, 3H), 3.25 (dd, *J* = 12.7, 6.5 Hz, 2H), 2.34 (t, *J* = 7.4 Hz, 2H), 1.76 (p, *J* = 7.0 Hz, 2H).

4-{[4-(6-Benzoylamino-benzo[1,3]dioxol-5-yl)-benzoylamino]-methyl}-benzoic acid methyl ester **(fb-1)**. ^1^H NMR (400 MHz, DMSO) δ 9.82 (s, 1H), 9.11 (t, *J* = 5.8 Hz, 1H), 7.89 (dd, *J* = 16.4, 8.2 Hz, 4H), 7.77 (d, *J* = 7.3 Hz, 2H), 7.51 (t, *J* = 10.2 Hz, 3H), 7.44 (dd, *J* = 11.5, 8.0 Hz, 4H), 7.02 (d, *J* = 8.8 Hz, 2H), 6.12 (s, 2H), 4.53 (d, *J* = 5.7 Hz, 2H), 3.83 (s, 3H).

4-({4-[6-(4-Methoxy-benzoylamino)-benzo[1,3]dioxol-5-yl]-benzoylamino}-methyl)-benzoic acid methyl ester **(fb-2)**. ^1^H NMR (400 MHz, DMSO) δ 9.67 (s, 1H), 9.15 (t, *J* = 5.8 Hz, 1H), 7.89 (dd, *J* = 18.5, 8.2 Hz, 4H), 7.77 (d, *J* = 8.6 Hz, 2H), 7.46 (dd, *J* = 23.8, 8.2 Hz, 4H), 6.99 (dd, *J* = 11.8, 7.1 Hz, 4H), 6.12 (s, 2H), 4.53 (d, *J* = 5.7 Hz, 2H), 3.84 (s, 3H), 3.79 (s, 3H).

4-({4-[6-(4-Ethyl-benzoylamino)-benzo[1,3]dioxol-5-yl]-benzoylamino}-methyl)-benzoic acid methyl ester **(fb-3)**. ^1^H NMR (400 MHz, DMSO) δ 9.74 (s, 1H), 9.12 (t, *J* = 5.8 Hz, 1H), 7.89 (dd, *J* = 17.5, 8.2 Hz, 4H), 7.70 (d, *J* = 7.9 Hz, 2H), 7.50 (d, *J* = 8.2 Hz, 2H), 7.43 (d, *J* = 8.1 Hz, 2H), 7.28 (d, *J* = 8.0 Hz, 2H), 7.01 (d, *J* = 6.6 Hz, 2H), 6.12 (s, 2H), 4.53 (d, *J* = 5.6 Hz, 2H), 3.83 (s, 3H), 2.63 (q, *J* = 7.5 Hz, 2H), 1.17 (t, *J* = 7.6 Hz, 3H).

4-({4-[6-(2-Fluoro-benzoylamino)-benzo[1,3]dioxol-5-yl]-benzoylamino}-methyl)-benzoic acid methyl ester **(fb-4)**. ^1^H NMR (400 MHz, DMSO) δ 9.75 (s, 1H), 9.16 (t, *J* = 5.7 Hz, 1H), 7.93 (d, *J* = 8.2 Hz, 4H), 7.52 (d, *J* = 7.9 Hz, 4H), 7.45 (d, *J* = 8.1 Hz, 2H), 7.26 (t, *J* = 9.9 Hz, 2H), 7.12 (s, 1H), 6.99 (s, 1H), 6.12 (s, 2H), 4.56 (d, *J* = 5.7 Hz, 2H), 3.84 (s, 3H).

4-({4-[6-(2-Trifluoromethyl-benzoylamino)-benzo[1,3]dioxol-5-yl]-benzoylamino}-methyl)-benzoic acid methyl ester **(fb-5)**. ^1^H NMR (400 MHz, DMSO) δ 10.01 (s, 1H), 9.18 (t, *J* = 5.8 Hz, 1H), 7.95 (dd, *J* = 12.7, 8.2 Hz, 4H), 7.79 (d, *J* = 7.8 Hz, 1H), 7.72 (t, *J* = 7.5 Hz, 1H), 7.64 (t, *J* = 7.6 Hz, 1H), 7.52 (d, *J* = 8.2 Hz, 2H), 7.48 − 7.39 (m, 3H), 6.99 (d, *J* = 4.4 Hz, 2H), 6.13 (s, 2H), 4.57 (d, *J* = 5.7 Hz, 2H), 3.84 (s, 3H).

4-({4-[6–(3-Fluoro-benzoylamino)-benzo[1,3]dioxol-5-yl]-benzoylamino}-methyl)-benzoic acid methyl ester **(fb-6)**. ^1^H NMR (400 MHz, DMSO) δ 9.93 (s, 1H), 9.12 (t, *J* = 5.7 Hz, 1H), 7.90 (dd, *J* = 14.6, 8.2 Hz, 4H), 7.63 (d, *J* = 7.6 Hz, 1H), 7.53 (dd, *J* = 27.3, 9.0 Hz, 4H), 7.40 (dd, *J* = 22.2, 8.1 Hz, 3H), 7.03 (d, *J* = 11.0 Hz, 2H), 6.13 (s, 2H), 4.54 (d, *J* = 5.7 Hz, 2H), 3.84 (s, 3H).

4-({4-[6-(4-Trifluoromethyl-benzoylamino)-benzo[1,3]dioxol-5-yl]-benzoylamino}-methyl)-benzoic acid methyl ester **(fb-7)**. ^1^H NMR (400 MHz, DMSO) δ 9.70 (s, 1H), 9.16 (s, 1H), 7.93 (d, *J* = 8.1 Hz, 4H), 7.50 (d, *J* = 8.2 Hz, 2H), 7.44 (d, *J* = 8.1 Hz, 2H), 7.30 (dd, *J* = 14.3, 7.2 Hz, 2H), 7.21 (d, *J* = 7.2 Hz, 2H), 7.05 (s, 1H), 6.98 (s, 1H), 6.12 (s, 2H), 4.57 (d, *J* = 5.7 Hz, 2H), 3.84 (s, 3H).

4-({4-[6-(3-Trifluoromethyl-benzoylamino)-benzo[1,3]dioxol-5-yl]-benzoylamino}-methyl)-benzoic acid methyl ester **(fb-8)**. ^1^H NMR (400 MHz, DMSO) δ 10.10 (s, 1H), 9.11 (t, *J* = 5.8 Hz, 1H), 8.08 (d, *J* = 8.4 Hz, 2H), 7.90 (dd, *J* = 12.8, 8.2 Hz, 5H), 7.71 (t, *J* = 7.7 Hz, 1H), 7.51 (d, *J* = 8.1 Hz, 2H), 7.42 (d, *J* = 8.1 Hz, 2H), 7.09 (s, 1H), 7.02 (s, 1H), 6.13 (s, 2H), 4.54 (d, *J* = 5.6 Hz, 2H), 3.84 (s, 3H).

4-({4-[6–(2-Methyl-benzoylamino)-benzo[1,3]dioxol-5-yl]-benzoylamino}-methyl)-benzoic acid methyl ester **(fb-9)**. ^1^H NMR (400 MHz, DMSO) δ 9.70 (s, 1H), 9.16 (s, 1H), 7.93 (d, *J* = 8.1 Hz, 4H), 7.47 (dd, *J* = 23.9, 8.1 Hz, 4H), 7.30 (dd, *J* = 14.3, 7.2 Hz, 2H), 7.21 (d, *J* = 7.2 Hz, 2H), 7.05 (s, 1H), 6.98 (s, 1H), 6.12 (s, 2H), 4.57 (d, *J* = 5.7 Hz, 2H), 3.84 (s, 3H), 2.20 (s, 3H).

4-({4-[6-(3-Bromo-benzoylamino)-benzo[1,3]dioxol-5-yl]-benzoylamino}-methyl)-benzoic acid methyl ester **(fb-10)**. ^1^H NMR (400 MHz, DMSO) δ 10.01 (s, 1H), 9.19 (t, *J* = 5.8 Hz, 1H), 7.91 (dd, *J* = 15.5, 7.7 Hz, 5H), 7.76 (dd, *J* = 21.8, 7.7 Hz, 2H), 7.45 (dt, *J* = 14.0, 7.2 Hz, 5H), 7.02 (d, *J* = 7.0 Hz, 2H), 6.13 (s, 2H), 4.53 (d, *J* = 5.7 Hz, 2H), 3.84 (s, 3H).

4-({4-[6–(4-Fluoro-benzoylamino)-benzo[1,3]dioxol-5-yl]-benzoylamino}-methyl)-benzoic acid methyl ester **(fb-11)**. ^1^H NMR (400 MHz, DMSO) δ 9.91 (d, *J* = 9.0 Hz, 1H), 9.20 (d, *J* = 5.9 Hz, 1H), 7.90 (dd, *J* = 11.1, 8.5 Hz, 6H), 7.49 (d, *J* = 8.0 Hz, 2H), 7.43 (d, *J* = 8.1 Hz, 2H), 7.29 (t, *J* = 8.8 Hz, 2H), 7.01 (d, *J* = 4.8 Hz, 2H), 6.12 (s, 2H), 4.53 (d, *J* = 5.7 Hz, 2H), 3.84 (s, 3H).

4-({4-[6–(2-Chloro-benzoylamino)-benzo[1,3]dioxol-5-yl]-benzoylamino}-methyl)-benzoic acid methyl ester **(fb-12)**. ^1^H NMR (400 MHz, DMSO) δ 9.92 (s, 1H), 9.22 (t, *J* = 5.6 Hz, 1H), 7.95 (dd, *J* = 10.6, 8.5 Hz, 4H), 7.52 (d, *J* = 8.2 Hz, 2H), 7.48 − 7.42 (m, 4H), 7.39 − 7.33 (m, 2H), 7.07 (s, 1H), 6.99 (s, 1H), 6.13 (s, 2H), 4.57 (d, *J* = 5.7 Hz, 2H), 3.84 (s, 3H).

4-{[4-(6-tert-Butoxycarbonylamino-benzo[1,3]dioxol-5-yl)-benzoylamino]-methyl}-benzoic acid methyl ester **(fb-13)**. ^1^H NMR (400 MHz, DMSO) δ 9.16 (t, *J* = 5.8 Hz, 1H), 8.37 (s, 1H), 7.93 (d, *J* = 7.8 Hz, 4H), 7.47 − 7.39 (m, 4H), 6.89 (d, *J* = 16.6 Hz, 2H), 6.07 (s, 2H), 4.57 (d, *J* = 5.7 Hz, 2H), 3.84 (s, 3H), 1.28 (s, 9H).

4-{[4-(6-Amino-benzo[1,3]dioxol-5-yl)-benzoylamino]-methyl}-benzoic acid methyl ester **(fb-14)**. ^1^H NMR (400 MHz, DMSO) δ 9.15 (t, *J* = 5.9 Hz, 1H), 7.95 (t, *J* = 7.8 Hz, 4H), 7.48 (dd, *J* = 20.3, 8.1 Hz, 4H), 6.64 (s, 1H), 6.45 (s, 1H), 5.89 (s, 2H), 4.64 (s, 2H), 4.58 (d, *J* = 5.9 Hz, 2H), 3.84 (s, 3H).

Compound **fa-1** (0.4 g, 0.74 mmol) was dissolved in NH_2_OK methanol solution. After reacting for about 1 h, the solvent was evaporated *in vacuo*. The residue was acidified with saturated citric acid and then extracted with EtOAc (3 × 20 ml). The organic layers were combined, washed with brine (3 × 20 ml), and dried with MgSO_4_. By crystallisation in EtOAc as a white powder, the desired compound **Fa-1** (0.33 g, 82% yield) was obtained.

4-{4-[6–(3-Bromo-benzoylamino)-benzo[1,3]dioxol-5-yl]-benzoylamino}-N-Hydroxy-butyramide **(Fa-1)**. ^1^H NMR (400 MHz, DMSO) δ 10.36 (s, 1H), 9.94 (s, 1H), 8.69 (s, 1H), 8.46 (t, *J* = 5.5 Hz, 1H), 7.94 (s, 1H), 7.81 (d, *J* = 8.4 Hz, 2H), 7.79 − 7.70 (m, 2H), 7.50 − 7.39 (m, 3H), 7.01 (d, *J* = 11.1 Hz, 2H), 6.12 (s, 2H), 3.23 (dd, *J* = 12.7, 6.7 Hz, 2H), 1.99 (t, *J* = 7.5 Hz, 2H), 1.78 − 1.66 (m, 2H).

### Intermediate Fa-2 through Fa-23, Fb-1 through Fb-14 were prepared as described for Fa-1

4-{4-[6–(2-Methoxy-benzoylamino)-benzo[1,3]dioxol-5-yl]-benzoylamino}-N-Hydroxy-butyramide **(Fa-2)**. ^1^H NMR (400 MHz, DMSO) δ 10.40 (d, *J* = 1.0 Hz, 1H), 9.77 (s, 1H), 8.71 (d, *J* = 1.6 Hz, 1H), 8.57 (t, *J* = 5.5 Hz, 1H), 7.98 − 7.90 (m, 3H), 7.84 − 7.77 (m, 1H), 7.54 − 7.45 (m, 3H), 7.07 (t, *J* = 7.5 Hz, 2H), 6.90 (s, 1H), 6.10 (d, *J* = 5.7 Hz, 2H), 3.40 (s, 3H), 3.27 (dd, *J* = 12.7, 6.7 Hz, 2H), 2.02 (t, *J* = 7.5 Hz, 2H), 1.81 − 1.71 (m, 2H).

4-{4-[6-(4-Fluoro-benzoylamino)-benzo[1,3]dioxol-5-yl]-benzoylamino}-N-Hydroxy-butyramide **(Fa-3)**. ^1^H NMR (400 MHz, DMSO) δ 10.19 (d, *J* = 140.2 Hz, 1H), 9.86 − 9.49 (m, 1H), 8.69 (s, 1H), 8.46 (t, *J* = 5.2 Hz, 1H), 7.85 (dd, *J* = 8.5, 5.7 Hz, 1H), 7.80 (dd, *J* = 8.5, 2.6 Hz, 2H), 7.66 (d, *J* = 8.7 Hz, 1H), 7.46 (dd, *J* = 8.4, 1.8 Hz, 2H), 7.29 (t, *J* = 8.8 Hz, 1H), 6.99 (t, *J* = 8.9 Hz, 2H), 6.77 (d, *J* = 8.7 Hz, 1H), 6.11 (d, *J* = 5.1 Hz, 2H), 3.22 (dd, *J* = 12.7, 6.7 Hz, 2H), 1.98 ((t, *J* = 7.6 Hz, 2H), 1.78 − 1.67 (m, 2H).

4-{4-[6-(2-Methyl-benzoylamino)-benzo[1,3]dioxol-5-yl]-benzoylamino}-N-Hydroxy-butyramide **(Fa-4)**. ^1^H NMR (400 MHz, DMSO) δ 10.38 (s, 1H), 9.68 (s, 1H), 8.70 (d, *J* = 1.2 Hz, 1H), 8.50 (t, *J* = 5.4 Hz, 1H), 7.87 (d, *J* = 8.3 Hz, 2H), 7.47 (d, *J* = 8.2 Hz, 2H), 7.29 (dd, *J* = 15.2, 7.7 Hz, 2H), 7.25 − 7.17 (m, 2H), 7.04 (s, 1H), 6.96 (s, 1H), 6.11 (s, 2H), 3.25 (dd, *J* = 12.6, 6.6 Hz, 2H), 2.20 (s, 3H), 2.01 (t, *J* = 7.5 Hz, 2H), 1.80 − 1.69 (m, 2H).

4-{4-[6-(3-Fluoro-benzoylamino)-benzo[1,3]dioxol-5-yl]-benzoylamino}-N-Hydroxy-butyramide **(Fa-5)**. ^1^H NMR (400 MHz, DMSO) δ 10.36 (s, 1H), 10.00 (d, *J* = 68.8 Hz, 1H), 8.69 (s, 1H), 8.45 (t, *J* = 5.0 Hz, 1H), 8.12 − 8.03 (m, 1H), 7.90 (t, *J* = 9.0 Hz, 1H), 7.81 (d, *J* = 8.3 Hz, 2H), 7.72 (t, *J* = 7.8 Hz, 1H), 7.59 − 7.34 (m, 3H), 7.05 (s, 1H), 7.01 (t, *J* = 5.2 Hz, 1H), 6.13 (d, *J* = 2.0 Hz, 2H), 3.22 (dd, *J* = 12.7, 6.4 Hz, 2H), 1.99 ((t, *J* = 7.4 Hz, 2H), 1.77 − 1.66 (m, 2H).

4-{4-[6-(2-Trifluoromethyl-benzoylamino)-benzo[1,3]dioxol-5-yl]-benzoylamino}-N-Hydroxy-butyramide **(Fa-6)**. ^1^H NMR (400 MHz, DMSO) δ 10.38 (s, 1H), 9.98 (s, 1H), 8.70 (d, *J* = 1.4 Hz, 1H), 8.52 (t, *J* = 5.5 Hz, 1H), 7.90 (d, *J* = 8.4 Hz, 2H), 7.79 (d, *J* = 7.7 Hz, 1H), 7.72 (t, *J* = 7.3 Hz, 1H), 7.64 (t, *J* = 7.6 Hz, 1H), 7.49 (d, *J* = 8.4 Hz, 2H), 7.40 (d, *J* = 7.5 Hz, 1H), 6.97 (d, *J* = 2.9 Hz, 2H), 6.12 (s, 2H), 3.26 (dd, *J* = 12.8, 6.8 Hz, 2H), 2.01 (t, *J* = 7.5 Hz, 2H), 1.75 (p, *J* = 7.3 Hz, 2H).

4-{4-[6-(4-Ethyl-benzoylamino)-benzo[1,3]dioxol-5-yl]-benzoylamino}-N-Hydroxy-butyramide **(Fa-7)**. ^1^H NMR (400 MHz, DMSO) δ 10.36 (s, 1H), 9.71 (s, 1H), 8.68 (s, 1H), 8.46 (t, *J* = 5.5 Hz, 1H), 7.80 (d, *J* = 8.4 Hz, 2H), 7.70 (d, *J* = 8.1 Hz, 2H), 7.46 (d, *J* = 8.4 Hz, 2H), 7.28 (d, *J* = 8.2 Hz, 2H), 7.00 (d, *J* = 10.4 Hz, 2H), 6.12 (s, 2H), 3.22 (dd, *J* = 12.7, 6.7 Hz, 2H), 2.64 (q, *J* = 7.6 Hz, 2H), 1.99 (t, *J* = 7.6 Hz, 2H), 1.72 (p, *J* = 7.2 Hz, 2H),1.23 − 1.13 (m, 3H).

4-{4-[6-(2-Fluoro-benzoylamino)-benzo[1,3]dioxol-5-yl]-benzoylamino}-N-Hydroxy-butyramide **(Fa-8)**. ^1^H NMR (400 MHz, DMSO) δ 10.39 (s, 1H), 9.73 (d, *J* = 2.0 Hz, 1H), 8.70 (s, 1H), 8.52 (t, *J* = 5.5 Hz, 1H), 7.87 (d, *J* = 8.4 Hz, 2H), 7.57 − 7.45 (m, 4H), 7.30 − 7.23 (m, 2H), 7.12 (s, 1H), 6.97 (s, 1H), 6.12 (s, 2H), 3.25 (dd, *J* = 12.7, 6.7 Hz, 2H), 2.01 (dd, *J* = 12.2, 4.4 Hz, 2H), 1.80 − 1.69 (m, 2H).

4-{4-[6-(3-Trifluoromethyl-benzoylamino)-benzo[1,3]dioxol-5-yl]-benzoylamino}-N-Hydroxy-butyramide **(Fa-9)**. ^1^H NMR (400 MHz, DMSO) δ 10.34 (s, 1H), 10.06 (s, 1H), 8.66 (d, *J* = 1.4 Hz, 1H), 8.43 (t, *J* = 5.5 Hz, 1H), 8.13 − 8.02 (m, 2H), 7.90 (d, *J* = 7.6 Hz, 1H), 7.81 (d, *J* = 8.3 Hz, 2H), 7.71 (t, *J* = 7.7 Hz, 1H), 7.47 (d, *J* = 8.2 Hz, 2H), 7.05 (s, 1H), 7.00 (s, 1H), 6.13 (s, 2H), 3.22 (dd, *J* = 12.7, 6.6 Hz, 2H), 1.99 (t, *J* = 7.5 Hz, 2H), 1.77 − 1.66 (m, 2H).

4-{4-[6-(2-Chloro-benzoylamino)-benzo[1,3]dioxol-5-yl]-benzoylamino}-N-Hydroxy-butyramide **(Fa-10)**. ^1^H NMR (400 MHz, DMSO) δ 10.40 (s, 1H), 9.87 (s, 1H), 8.68 (d, *J* = 1.4 Hz, 1H), 8.52 (t, *J* = 5.1 Hz, 1H), 7.89 (d, *J* = 8.3 Hz, 2H), 7.52 − 7.46 (m, 3H), 7.46 − 7.33 (m, 3H), 7.06 (s, 1H), 6.97 (s, 1H), 6.12 (s, 2H), 3.26 (dd, *J* = 12.8, 6.7 Hz, 2H), 2.02 (t, *J* = 7.5 Hz, 2H), 1.75 (p, *J* = 7.3 Hz, 2H).

4-[4–(6-Benzoylamino-benzo[1,3]dioxol-5-yl)-benzoylamino]-N-Hydroxy-butyramide **(Fa-11)**. ^1^H NMR (400 MHz, DMSO) δ 10.34 (s, 1H), 9.78 (s, 1H), 8.66 (d, *J* = 1.6 Hz, 1H), 8.44 (t, *J* = 5.5 Hz, 1H), 7.79 (dd, *J* = 14.3, 7.9 Hz, 4H), 7.52 (t, *J* = 7.3 Hz, 1H), 7.49 − 7.41 (m, 4H), 7.02 (s, 1H), 6.98 (s, 1H), 6.12 (s, 2H), 3.22 (dd, *J* = 12.7, 6.7 Hz, 2H), 1.99 (t, *J* = 7.5 Hz, 2H), 1.77 − 1.67 (m, 2H).

4-{4-[6–(4-Trifluoromethyl-benzoylamino)-benzo[1,3]dioxol-5-yl]-benzoylamino}-N-Hydroxy-butyramide **(Fa-12)**. ^1^H NMR (400 MHz, DMSO) δ 10.34 (s, 1H), 10.04 (s, 1H), 8.66 (s, 1H), 8.43 (t, *J* = 5.5 Hz, 1H), 7.96 (d, *J* = 8.1 Hz, 2H), 7.82 (dd, *J* = 14.4, 8.4 Hz, 4H), 7.47 (d, *J* = 8.3 Hz, 2H), 7.04 (s, 1H), 7.00 (s, 1H), 6.12 (s, 2H), 3.22 (dd, *J* = 12.8, 6.7 Hz, 2H), 1.99 (t, *J* = 7.5 Hz, 2H), 1.72 (p, *J* = 7.3 Hz, 2H).

4-{4-[6–(4-Methoxy-benzoylamino)-benzo[1,3]dioxol-5-yl]-benzoylamino}-N-Hydroxy-butyramide **(Fa-13)**. ^1^H NMR (400 MHz, DMSO) δ 10.35 (s, 1H), 9.61 (s, 1H), 8.66 (s, 1H), 8.44 (t, *J* = 5.5 Hz, 1H), 7.80 (s, 1H), 7.79 − 7.74 (m, 3H), 7.46 (d, *J* = 8.4 Hz, 2H), 7.00 (s, 1H), 6.99 − 6.95 (m, 3H), 6.11 (s, 2H), 3.79 (s, 3H), 3.22 (dd, *J* = 12.8, 6.7 Hz, 2H), 2.03 − 1.95 (m, 2H), 1.78 − 1.67 (m, 2H).

4-{4-[6–(3,5-Difluoro-benzoylamino)-benzo[1,3]dioxol-5-yl]-benzoylamino}-N-Hydroxy-butyramide **(Fa-14)**. ^1^H NMR (400 MHz, DMSO) δ 10.35 (s, 1H), 9.98 (s, 1H), 8.66 (s, 1H), 8.45 (t, *J* = 5.5 Hz, 1H), 7.82 (d, *J* = 8.4 Hz, 2H), 7.46 (dd, *J* = 10.3, 5.6 Hz, 5H), 7.01 (d, *J* = 9.1 Hz, 2H), 6.12 (s, 2H), 3.23 (dd, *J* = 12.8, 6.7 Hz, 2H), 2.03 − 1.95 (m, 2H), 1.73 (p, *J* = 7.3 Hz, 2H).

4-{4-[6–(2,4-Difluoro-benzoylamino)-benzo[1,3]dioxol-5-yl]-benzoylamino}-N-Hydroxy-butyramide **(Fa-15)**. ^1^H NMR (400 MHz, DMSO) δ 10.36 (s, 1H), 9.70 (s, 1H), 8.67 (s, 1H), 8.49 (t, *J* = 5.4 Hz, 1H), 7.86 (d, *J* = 8.4 Hz, 2H), 7.61 (dd, *J* = 15.2, 8.4 Hz, 1H), 7.48 (d, *J* = 8.1 Hz, 2H), 7.37 − 7.27 (m, 1H), 7.17 (dd, *J* = 8.5, 2.3 Hz, 1H), 7.12 (s, 1H), 6.96 (s, 1H), 6.11 (s, 2H), 3.25 (dd, *J* = 12.8, 6.6 Hz, 2H), 2.02 (t, *J* = 7.5 Hz, 2H), 1.75 (p, *J* = 7.3 Hz, 2H).

4-{4-[6–(4-Trifluoromethoxy-benzoylamino)-benzo[1,3]dioxol-5-yl]-benzoylamino}-N-Hydroxy-butyramide **(Fa-16)**. ^1^H NMR (400 MHz, DMSO) δ 10.35 (s, 1H), 9.93 (s, 1H), 8.66 (s, 1H), 8.44 (t, *J* = 5.5 Hz, 1H), 7.90 (d, *J* = 8.6 Hz, 2H), 7.81 (d, *J* = 8.4 Hz, 2H), 7.46 (dd, *J* = 8.0, 5.1 Hz, 4H), 7.02 (s, 1H), 6.99 (s, 1H), 6.12 (s, 2H), 3.23 (dd, *J* = 12.8, 6.7 Hz, 2H), 2.04(t, *J* = 7.5 Hz, 2H), 1.73 (p, *J* = 7.3 Hz, 2H).

4-{4-[6–(3-Nitro-benzoylamino)-benzo[1,3]dioxol-5-yl]-benzoylamino}-N-Hydroxy-butyramide **(Fa-17)**. ^1^H NMR (400 MHz, DMSO) δ 10.35 (s, 1H), 9.78 (s, 1H), 8.66 (s, 1H), 8.44 (t, *J* = 5.4 Hz, 1H), 7.79 (dd, *J* = 14.5, 7.9 Hz, 4H), 7.49 − 7.43 (m, 4H), 7.02 (s, 1H), 6.98 (s, 1H), 6.12 (s, 2H), 3.22 (dd, *J* = 12.5, 6.4 Hz, 2H), 2.03 − 1.96 (m, 2H), 1.73 (dd, *J* = 14.3, 7.1 Hz, 2H).

4-{4-[6–(4-Fluoro-2-trifluoromethyl-benzoylamino)-benzo[1,3]dioxol-5-yl]-benzoylamino}-N-Hydroxy-butyramide **(Fa-18)**. ^1^H NMR (400 MHz, DMSO) δ 10.37 (s, 1H), 9.98 (s, 1H), 8.67 (s, 1H), 8.50 (t, *J* = 5.5 Hz, 1H), 7.88 (t, *J* = 6.1 Hz, 2H), 7.71 (dd, *J* = 9.3, 2.5 Hz, 1H), 7.62 (td, *J* = 8.4, 2.4 Hz, 1H), 7.52 − 7.43 (m, 3H), 6.96 (dd, *J* = 12.4, 6.6 Hz, 2H), 6.12 (s, 2H), 3.26 (dd, *J* = 12.8, 6.7 Hz, 2H), 2.02 (t, *J* = 7.4 Hz, 2H), 1.75 (p, *J* = 7.3 Hz, 2H).

4-{4-[6–(3-Chloro-4-fluoro-benzoylamino)-benzo[1,3]dioxol-5-yl]-benzoylamino}-N-Hydroxy-butyramide **(Fa-19)**. ^1^H NMR (400 MHz, DMSO) δ 10.35 (s, 1H), 9.93 (s, 1H), 8.67 (s, 1H), 8.44 (t, *J* = 5.5 Hz, 1H), 7.98 (dd, *J* = 7.1, 1.8 Hz, 1H), 7.81 (dd, *J* = 8.4, 3.3 Hz, 3H), 7.52 (t, *J* = 8.9 Hz, 1H), 7.45 (d, *J* = 8.3 Hz, 2H), 7.02 (s, 1H), 6.99 (d, *J* = 2.6 Hz, 1H), 6.12 (s, 2H), 3.23 (dd, *J* = 12.8, 6.7 Hz, 2H), 2.04 (t, *J* = 7.6 Hz, 2H), 1.78 − 1.68 (m, 2H).

4-{4-[6–(4-Propyl-benzoylamino)-benzo[1,3]dioxol-5-yl]-benzoylamino}-N-Hydroxy-butyramide **(Fa-20)**. ^1^H NMR (400 MHz, DMSO) δ 10.35 (s, 1H), 9.69 (s, 1H), 8.67 (s, 1H), 8.44 (t, *J* = 5.5 Hz, 1H), 7.81 (d, *J* = 8.4 Hz, 2H), 7.70 (d, *J* = 8.0 Hz, 2H), 7.47 (d, *J* = 8.3 Hz, 2H), 7.26 (d, *J* = 8.2 Hz, 2H), 7.01 (s, 1H), 6.98 (s, 1H), 6.11 (s, 2H), 3.23 (dd, *J* = 12.7, 6.6 Hz, 2H), 2.58 (t, *J* = 7.5 Hz, 2H), 2.05 (t, *J* = 7.6 Hz, 2H), 1.78 − 1.67 (m, 2H), 1.65 − 1.53 (m, 2H), 0.88 (t, *J* = 7.3 Hz, 3H).

4-{4-[6–(2-Chloro-6-fluoro-benzoylamino)-benzo[1,3]dioxol-5-yl]-benzoylamino}-N-Hydroxy-butyramide **(Fa-21)**. ^1^H NMR (400 MHz, DMSO) δ 10.38 (s, 1H), 10.14 (s, 1H), 8.68 (s, 1H), 8.50 (t, *J* = 5.5 Hz, 1H), 7.87 (d, *J* = 8.3 Hz, 2H), 7.52 − 7.42 (m, 3H), 7.35 (d, *J* = 8.0 Hz, 1H), 7.29 (dd, *J* = 11.0, 6.0 Hz, 1H), 7.04 (s, 1H), 6.98 (s, 1H), 6.12 (s, 2H), 3.26 (dd, *J* = 12.8, 6.7 Hz, 2H), 2.02 (t, *J* = 7.5 Hz, 2H), 1.81 − 1.70 (m, 2H).

4-{4-[6–(2,5-Difluoro-benzoylamino)-benzo[1,3]dioxol-5-yl]-benzoylamino}-N-Hydroxy-butyramide **(Fa-22)**. ^1^H NMR (400 MHz, DMSO) δ 10.36 (s, 1H), 9.80 (d, *J* = 1.3 Hz, 1H), 8.67 (s, 1H), 8.49 (t, *J* = 5.5 Hz, 1H), 7.87 (d, *J* = 8.4 Hz, 2H), 7.48 (d, *J* = 8.3 Hz, 2H), 7.40 − 7.29 (m, 3H), 7.13 (s, 1H), 6.97 (s, 1H), 6.12 (s, 2H), 3.25 (dd, *J* = 12.9, 6.7 Hz, 2H), 2.01 (t, *J* = 7.5 Hz, 2H), 1.75 (p, *J* = 7.3 Hz, 2H).

4-{4-[6–(4-Fluoro-3-trifluoromethyl-benzoylamino)-benzo[1,3]dioxol-5-yl]-benzoylamino}-N-Hydroxy-butyramide **(Fa-23)**. ^1^H NMR (400 MHz, DMSO) δ 10.35 (s, 1H), 9.77 (s, 1H), 8.67 (s, 1H), 8.43 (t, *J* = 5.5 Hz, 1H), 7.99 (s, 1H), 7.89 (d, *J* = 8.5 Hz, 1H), 7.80 (d, *J* = 8.4 Hz, 2H), 7.45 (d, *J* = 8.4 Hz, 2H), 7.05 (d, *J* = 8.6 Hz, 1H), 7.00 (s, 1H), 6.98 (s, 1H), 6.11 (s, 2H), 3.23 (dd, *J* = 12.7, 6.7 Hz, 2H), 2.00 (t, *J* = 7.5 Hz, 2H), 1.78 − 1.68 (m, 2H).

4-{[4–(6-Benzoylamino-benzo[1,3]dioxol-5-yl)-benzoylamino]-methyl}-N-Hydroxy-benzamide **(Fb-1)**. ^1^H NMR (400 MHz, DMSO) δ 11.16 (s, 1H), 9.82 (s, 2H), 9.80 − 9.45 (m, 1H), 9.08 (t, *J* = 5.7 Hz, 1H), 7.87 (d, *J* = 8.2 Hz, 2H), 7.77 (d, *J* = 7.4 Hz, 2H), 7.69 (d, *J* = 8.1 Hz, 2H), 7.56 − 7.40 (m, 5H), 7.35 (d, *J* = 8.1 Hz, 2H), 7.02 (d, *J* = 10.0 Hz, 2H), 6.94 − 6.93 (m, 1H), 6.12 (s, 2H).

4-({4-[6–(4-Methoxy-benzoylamino)-benzo[1,3]dioxol-5-yl]-benzoylamino}-methyl)-N-Hydroxy-benzamide **(Fb-2)**. ^1^H NMR (400 MHz, DMSO) δ 11.16 (s, 1H), 9.65 (s, 1H), 9.07 (t, *J* = 5.6 Hz, 2H), 7.87 (t, *J* = 11.7 Hz, 2H), 7.77 (d, *J* = 8.6 Hz, 2H), 7.69 (d, *J* = 8.1 Hz, 2H), 7.48 (d, *J* = 8.2 Hz, 2H), 7.36 (t, *J* = 9.2 Hz, 2H), 6.99 (dd, *J* = 11.4, 7.9 Hz, 4H), 6.12 (s, 2H), 4.49 (d, *J* = 5.5 Hz, 2H), 3.79 (s, 3H).

4-({4-[6–(4-Ethyl-benzoylamino)-benzo[1,3]dioxol-5-yl]-benzoylamino}-methyl)-N-Hydroxy-benzamide **(Fb-3)**. ^1^H NMR (400 MHz, DMSO) δ 11.16 (s, 1H), 9.73 (s, 1H), 9.08 (t, *J* = 5.5 Hz, 1H), 9.03 − 8.81 (m, 1H), 7.86 (d, *J* = 8.1 Hz, 2H), 7.74 − 7.65 (m, 4H), 7.49 (d, *J* = 8.0 Hz, 2H), 7.35 (d, *J* = 7.9 Hz, 2H), 7.28 (d, *J* = 7.9 Hz, 2H), 7.01 (d, *J* = 7.5 Hz, 2H), 6.12 (s, 2H), 4.49 (d, *J* = 5.5 Hz, 2H), 2.69 − 2.59 (m, 2H), 1.17 (t, *J* = 7.5 Hz, 3H).

### 4-({4-[6–(2-Fluoro-benzoylamino)-benzo[1,3]dioxol-5-yl]-benzoylamino}-methyl)-N-Hydroxy-benzamide (Fb-4)

^1^H NMR (400 MHz, DMSO) δ 11.17 (s, 1H), 9.75 (s, 1H), 9.13 (t, *J* = 5.7 Hz, 1H), 9.01 (s, 1H), 7.93 (d, *J* = 8.2 Hz, 2H), 7.71 (d, *J* = 8.1 Hz, 2H), 7.52 (t, *J* = 7.8 Hz, 4H), 7.37 (d, *J* = 8.1 Hz, 2H), 7.26 (t, *J* = 9.9 Hz, 2H), 7.12 (s, 1H), 6.99 (s, 1H), 6.12 (s, 2H), 4.52 (d, *J* = 5.6 Hz, 2H).

### 4-({4-[6–(2-Trifluoromethyl-benzoylamino)-benzo[1,3]dioxol-5-yl]-benzoylamino}-methyl)-N-Hydroxy-benzamide (Fb-5)

^1^H NMR (400 MHz, DMSO) δ 11.17 (s, 1H), 10.00 (s, 1H), 9.14 (t, *J* = 5.7 Hz, 1H), 9.00 (s, 1H), 7.96 (d, *J* = 8.2 Hz, 2H), 7.79 (d, *J* = 7.7 Hz, 1H), 7.71 (d, *J* = 8.0 Hz, 3H), 7.64 (t, *J* = 7.5 Hz, 1H), 7.51 (d, *J* = 8.2 Hz, 2H), 7.45 − 7.39 (m, 1H), 7.37 (d, *J* = 8.1 Hz, 2H), 6.99 (d, *J* = 3.4 Hz, 2H), 6.13 (s, 2H), 4.53 (d, *J* = 5.6 Hz, 2H).

### 4-({4-[6–(3-Fluoro-benzoylamino)-benzo[1,3]dioxol-5-yl]-benzoylamino}-methyl)-N-Hydroxy-benzamide (Fb-6)

^1^H NMR (400 MHz, DMSO) δ 10.37 (s, 1H), 9.98 (s, 1H), 8.67 (s, 1H), 8.50 (t, *J* = 5.5 Hz, 1H), 7.88 (t, *J* = 6.6 Hz, 2H), 7.71 (dd, *J* = 9.3, 2.5 Hz, 1H), 7.62 (td, *J* = 8.4, 2.4 Hz, 1H), 7.52 − 7.43 (m, 3H), 6.96 (dd, *J* = 12.4, 6.6 Hz, 2H), 6.11 (d, *J* = 4.6 Hz, 2H), 3.26 (dd, *J* = 12.8, 6.7 Hz, 2H), 2.02 (t, *J* = 7.4 Hz, 2H), 1.75 (p, *J* = 7.3 Hz, 2H).

### 4-({4-[6–(4-Trifluoromethyl-benzoylamino)-benzo[1,3]dioxol-5-yl]-benzoylamino}-methyl)-N-Hydroxy-benzamide (Fb-7)

^1^H NMR (400 MHz, DMSO) δ 11.16 (s, 1H), 10.08 (s, 1H), 9.06 (d, *J* = 5.8 Hz, 1H), 9.03 − 8.88 (m, 1H), 7.96 (d, *J* = 7.9 Hz, 2H), 7.86 (t, *J* = 8.0 Hz, 4H), 7.69 (d, *J* = 8.0 Hz, 2H), 7.49 (d, *J* = 8.0 Hz, 2H), 7.35 (d, *J* = 8.0 Hz, 2H), 7.04 (d, *J* = 13.3 Hz, 2H), 6.13 (s, 2H), 4.49 (d, *J* = 5.5 Hz, 2H).

### 4-({4-[6–(3-Trifluoromethyl-benzoylamino)-benzo[1,3]dioxol-5-yl]-benzoylamino}-methyl)-N-Hydroxy-benzamide (Fb-8)

^1^H NMR (400 MHz, DMSO) δ 11.16 (s, 1H), 10.09 (s, 1H), 9.07 (t, *J* = 5.7 Hz, 1H), 8.99 (s, 1H), 8.07 (d, *J* = 10.6 Hz, 2H), 7.89 (dd, *J* = 15.9, 8.1 Hz, 3H), 7.71 (dd, *J* = 13.7, 8.0 Hz, 3H), 7.50 (d, *J* = 8.1 Hz, 2H), 7.34 (d, *J* = 8.1 Hz, 2H), 7.04 (d, *J* = 17.4 Hz, 2H), 6.13 (s, 2H), 4.49 (d, *J* = 5.6 Hz, 2H).

### 4-({4-[6–(2-Methyl-benzoylamino)-benzo[1,3]dioxol-5-yl]-benzoylamino}-methyl)-N-Hydroxy-benzamide (Fb-9)

^1^H NMR (400 MHz, DMSO) δ 11.17 (s, 1H), 9.70 (s, 1H), 9.12 (t, *J* = 5.6 Hz, 1H), 9.00 (s, 1H), 7.93 (d, *J* = 8.1 Hz, 2H), 7.71 (d, *J* = 8.0 Hz, 2H), 7.50 (d, *J* = 8.1 Hz, 2H), 7.36 (d, *J* = 8.0 Hz, 2H), 7.31 − 7.25 (m, 2H), 7.24 − 7.17 (m, 2H), 7.05 (s, 1H), 6.98 (s, 1H), 6.12 (s, 2H), 4.52 (d, *J* = 5.5 Hz, 2H), 2.20 (s, 3H).

### 4-({4-[6–(3-Bromo-benzoylamino)-benzo[1,3]dioxol-5-yl]-benzoylamino}-methyl)-N-Hydroxy-benzamide (Fb-10)

^1^H NMR (400 MHz, DMSO) δ 11.17 (s, 1H), 9.91 (s, 1H), 9.13 (t, *J* = 5.7 Hz, 1H), 9.00 (s, 1H), 7.94 (d, *J* = 8.2 Hz, 2H), 7.71 (d, *J* = 8.1 Hz, 2H), 7.55 − 7.49 (m, 2H), 7.48 (s, 1H), 7.46 − 7.40 (m, 1H), 7.37 (d, *J* = 6.0 Hz, 4H), 7.07 (s, 1H), 6.99 (s, 1H), 6.12 (s, 2H), 4.53 (d, *J* = 5.6 Hz, 2H).

### 4-({4-[6–(4-Fluoro-benzoylamino)-benzo[1,3]dioxol-5-yl]-benzoylamino}-methyl)-N-Hydroxy-benzamide (Fb-11)

^1^H NMR (400 MHz, DMSO) δ 11.16 (s, 1H), 9.95 (s, 1H), 9.08 (t, *J* = 5.5 Hz, 1H), 8.99 (s, 1H), 7.93 (s, 1H), 7.88 (d, *J* = 8.2 Hz, 2H), 7.81 − 7.65 (m, 4H), 7.47 (t, *J* = 7.2 Hz, 2H), 7.43 (t, *J* = 7.8 Hz, 1H), 7.35 (d, *J* = 8.0 Hz, 2H), 7.02 (d, *J* = 9.6 Hz, 2H), 6.13 (s, 2H), 4.49 (d, *J* = 5.5 Hz, 2H).

### 4-({4-[6–(2-Chloro-benzoylamino)-benzo[1,3]dioxol-5-yl]-benzoylamino}-methyl)-N-Hydroxy-benzamide (Fb-12)

^1^H NMR (400 MHz, DMSO) δ 11.17 (s, 1H), 9.91 (s, 1H), 9.13 (t, *J* = 5.5 Hz, 1H), 9.00 (s, 1H), 7.93 (t, *J* = 10.3 Hz, 2H), 7.71 (d, *J* = 8.1 Hz, 2H), 7.56 − 7.49 (m, 2H), 7.47 (d, *J* = 7.2 Hz, 1H), 7.46 − 7.39 (m, 1H), 7.37 (d, *J* = 5.8 Hz, 4H), 7.07 (s, 1H), 6.99 (s, 1H), 6.12 (s, 2H), 4.53 (d, *J* = 5.6 Hz, 2H).

### 4-{[4–(6-Tert-Butoxycarbonylamino-benzo[1,3]dioxol-5-yl)-benzoylamino]-methyl}-N-Hydroxy-benzamide (Fb-13)

^1^H NMR (400 MHz, DMSO) δ 11.17 (s, 1H), 9.11 (d, *J* = 5.9 Hz, 1H), 8.99 (s, 1H), 8.36 (s, 1H), 7.92 (d, *J* = 8.2 Hz, 2H), 7.71 (d, *J* = 8.2 Hz, 2H), 7.43 (d, *J* = 8.1 Hz, 2H), 7.36 (d, *J* = 8.1 Hz, 2H), 6.90 (s, 1H), 6.86 (s, 1H), 6.07 (s, 2H), 4.53 (d, *J* = 5.8 Hz, 2H), 1.27 (s, 9H).

### 4-{[4–(6-Amino-benzo[1,3]dioxol-5-yl)-benzoylamino]-methyl}-N-Hydroxy-benzamide (Fb-14)

^1^H NMR (400 MHz, DMSO) δ 11.22 (s, 1H), 9.78 (s, 1H), 9.31 (t, *J* = 5.8 Hz, 1H), 8.03 (d, *J* = 8.0 Hz, 2H), 7.73 (d, *J* = 7.9 Hz, 2H), 7.64 (d, *J* = 8.0 Hz, 2H), 7.38 (d, *J* = 8.0 Hz, 2H), 7.15 (s, 1H), 7.00 (s, 1H), 6.14 (s, 2H), 4.55 (d, *J* = 5.7 Hz, 2H), 3.62 (d, *J* = 180.5 Hz, 2H).

### *In vitro* HDACs inhibitory assay

All HDAC enzymes were purchased from BPS Bioscience. In short, 60 μL of recombinant HDAC enzyme solution was mixed with various concentrations of test compound (40 μL), and then incubated at 37 °C for 30 min. The reaction was terminated by adding 100 μL of imaging agent containing trypsin and trichostatin A (TSA). After standing for 20 min, the fluorescence intensity was measured at the excitation and emission wavelengths of 360 and 460 nm with a microplate reader. The inhibition rate was calculated from the fluorescence intensity readings of the test wells relative to the control wells, and the IC_50_ curve and value were determined by GraphPad Prism 6.0 software.

### *In vitro* antiproliferative activity

SAHA was used as a control and the MTT assay was used to determine tumour cell suppression. K562, U266, MCF-7, U937 and HEPG2 cells were cultured in corresponding medium supplemented with 10% FBS. Dilute the stock solution of the test compound with the culture medium. In short, cells were seeded into each well of a 96-well plate and incubated at 37 °C under 5% CO_2_. The cells were then treated with various concentrations of compound samples for 48 h. After that, add 10 μl MTT working solution to each well and incubate for another 4 h. After incubation, the medium formed by MTT was extracted by adding DMSO (100 µL). Measure the absorbance (OD) at 570 nm and 630 nm with a microplate reader. The cell growth inhibition rate was calculated according to the following formula:% inhibition = [1- (Sample group OD_570_ – Sample group OD_630_)/(Control group OD_570_ – Control group OD_630_)] × 100%. Use Origin 7.5 software to calculate IC50 value.

### Cell cycle assay

MCF-7 cells in the logarithmic growth phase were seeded in a 6-well plate and incubated with different doses of **Fb-4** and SAHA (1, 3, and 9 μM) for 24 h. The cells were then washed twice with cold PBS and fixed in 70% pre-chilled ethanol at 4 °C for 6 h. After fixation, the cells were washed again with PBS and stained with PI/RNase A at 37 °C for 30 min, and then stored at 4 °C in the dark. After staining, the cell cycle distribution was determined by flow cytometry within 24 h.

### Cell apoptosis assay

MCF-7 cells in the logarithmic growth phase were seeded in 6-well plates and incubated with different doses of **Fb-4** and SAHA (1, 3 and 9 µM) for 24 h. After that, wash the cells with PBS and collect the cells, resuspend them in the binding buffer of the Annexin V-FITC kit, then add 5 μL of Annexin V-FITC and mix gently, and then place it at 2–8 °C in the dark Incubate. After incubating for 15 min, add 10 µL of PI to each sample and mix gently, and incubate for 5 min at 2–8 °C under dark conditions, and detect with a flow cytometer.
